# Rational Design of Inner Ear Drug Delivery Systems

**DOI:** 10.1002/advs.202410568

**Published:** 2025-05-08

**Authors:** Xiayidan Maimaitikelimu, Zhiyan Xuan, Haoyu Ren, Keng Chen, Hui Zhang, Huan Wang

**Affiliations:** ^1^ The Eighth Affiliated Hospital Sun Yat‐Sen University Shenzhen 518033 China; ^2^ School of Life Sciences and Technology Southeast University Nanjing 210000 China

**Keywords:** drug delivery, hair cells, hearing loss, inner ear, physiologic barriers

## Abstract

The number of people with hearing loss disorders is enormous, causing great physical and mental stress to patients, as well as a huge social burden. Among these patients, hearing loss caused by inner ear lesions accounts for a large proportion. Therefore, treatment of the inner ear is important. Inner ear drug delivery systems, which can reduce the side effects of systemic drug administration by delivering drugs directly to the inner ear, are important in sensorineural hearing loss. Here, the development of inner ear drug delivery systems is focused, including the complex physiological structure that they face, types of drugs delivered, routes of administration, and forms of drug delivery carrier platforms. Recent studies in this process are presented and it is concluded with a summary and outlook on the problems faced and possible solutions.

## Graphical abstract

Hearing loss is a common disease affecting many people, and inner ear lesions are one of the most important causes. This review focuses on the treatment of inner ear hearing loss by drug delivery systems. It includes the current methods and technologies developed, and it predicts possible directions.

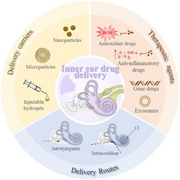

## 1 Introduction

The World Health Organization has reported that more than 1.5 billion people are currently affected by hearing loss worldwide.^[^ ^1^ ^]^ This number is expected to increase to nearly 2.5 billion by 2050, meaning that one‐quarter of the world's population will be affected by some degree of hearing loss, and ≈403 million people require hearing rehabilitation services.^[^ ^2^ ^]^ Hearing loss affects all age groups and is particularly prevalent among older people.^[^ ^3^ ^]^ Still, children and adolescents with more fragile hearing systems are also at risk of hearing damage due to inappropriate use of headphones or exposure to high‐intensity noise.^[^ ^4^ ^]^ Hearing loss directly contributes to communication difficulties and affects the interactions in school, work, and social situations.^[^ ^5^ ^]^ Long‐term hearing loss can lead to socioemotional deficits, which results in mental health problems such as loss of self‐confidence, anxiety, depression, and even social isolation.^[^ ^6^ ^]^ Moreover, hearing loss is strongly associated with cognitive decline and may increase the risk of developing dementia, such as Alzheimer's disease.^[^ ^7^ ^]^ Consequently, hearing loss can significantly reduce an individual's overall quality of life. From a societal perspective, as the population with hearing loss increases, the demand for related medical and rehabilitation services also increases, exerting pressure on the healthcare system. At the same time, hearing loss can affect employability, leading to increased unemployment or reduced work efficiency, thus negatively affecting the socioeconomic situation.^[^ ^8^ ^]^ In addition, children with hearing loss require more educational resources and support in their language development and learning. Therefore, hearing loss represents a significant societal burden.

Generally, hearing loss can be divided into conductive, sensorineural, and mixed types, with sensorineural hearing loss being the main cause. The sensorineural hearing loss is typically caused by functional lesions of the cochlea, auditory nerve, or auditory center, which result from the following reasons. First, the degenerative changes during natural aging result in gradual damage or functional loss of hair cells (HCs), spiral ganglion neurons (SGNs), and stria vascularis cells.^[^ ^9^ ^]^ Second, prolonged exposure to high noise levels induces irreversible mechanical damages to the Corti and the loss of HCs and SGNs.^[^ ^10^ ^]^ Third, the use of ototoxic drugs, including streptomycin and cisplatin, also causes the loss of HCs.^[^ ^11^ ^]^ Additionally, genetic factors are a significant contributor to sensorineural hearing loss.^[^ ^12^ ^]^ Genetic mutations not only result in structural or functional abnormalities in the auditory organs, subsequently impacting hearing directly, but also increase the susceptibility to damage caused by aging, noise, and ototoxic drugs. The complexity and diversity of the etiological factors contribute to treatment difficulties.

To date, cochlear implantation and systemic drug administration are the main methods used to treat sensorineural hearing loss.^[^ ^13^ ^]^ Cochlear implants (CIs) are electronic devices that partly replace the function of damaged inner ear HCs by converting sound signals into electrical signals and stimulating the auditory nerve directly, helping patients restore or acquire hearing.^[^ ^14^ ^]^ It is particularly appropriate for patients with severe sensorineural deafness and is currently recognized as an effective treatment worldwide.^[^ ^15^ ^]^ However, cochlear implantation has many limitations such as high cost, complex procedures, foreign body inflammation, and long‐term hearing and speech rehabilitation.^[^ ^16^ ^]^ Systemic drug delivery can be achieved through oral administration or injections. Drug administration alleviates or treats hearing loss by improving the microcirculation in the inner ear and nourishing nerves.^[^ ^17^ ^]^ This method is suitable for certain types of sensorineural hearing loss such as sudden deafness. Nevertheless, its therapeutic effect is influenced by a variety of factors, such as the type of drug, dosage, and individual patient differences, which may cause toxic side effects. In addition, the desired therapeutic effects are difficult to achieve in patients who have already suffered irreversible damage to inner ear HCs. Therefore, the specific situation of each patient should be comprehensively assessed before therapeutic strategies are implemented, and novel treatments are still anticipated.

Recently, biotechnology has made significant progress in drug delivery systems for the inner ear.^[^ ^18^ ^]^ Direct and accurate drug delivery to the inner ear tissue addresses numerous limitations of current drug therapy methods, thereby offering new avenues for the treatment of sensorineural hearing loss.^[^ ^19^ ^]^ Compared with oral or injection administration, the inner ear drug delivery system circumvents the limitations imposed by the blood labyrinth barrier, enabling the delivery of drugs to the lesion site at a high concentration and small loss, thereby improving the drug utilization and reducing systemic side effects.^[^ ^20^ ^]^ Furthermore, the advent of novel delivery routes has rendered drugs that were previously challenging to deliver using traditional methods paving the way for innovative drug development.^[^ ^21^ ^]^

**Figure 1:**
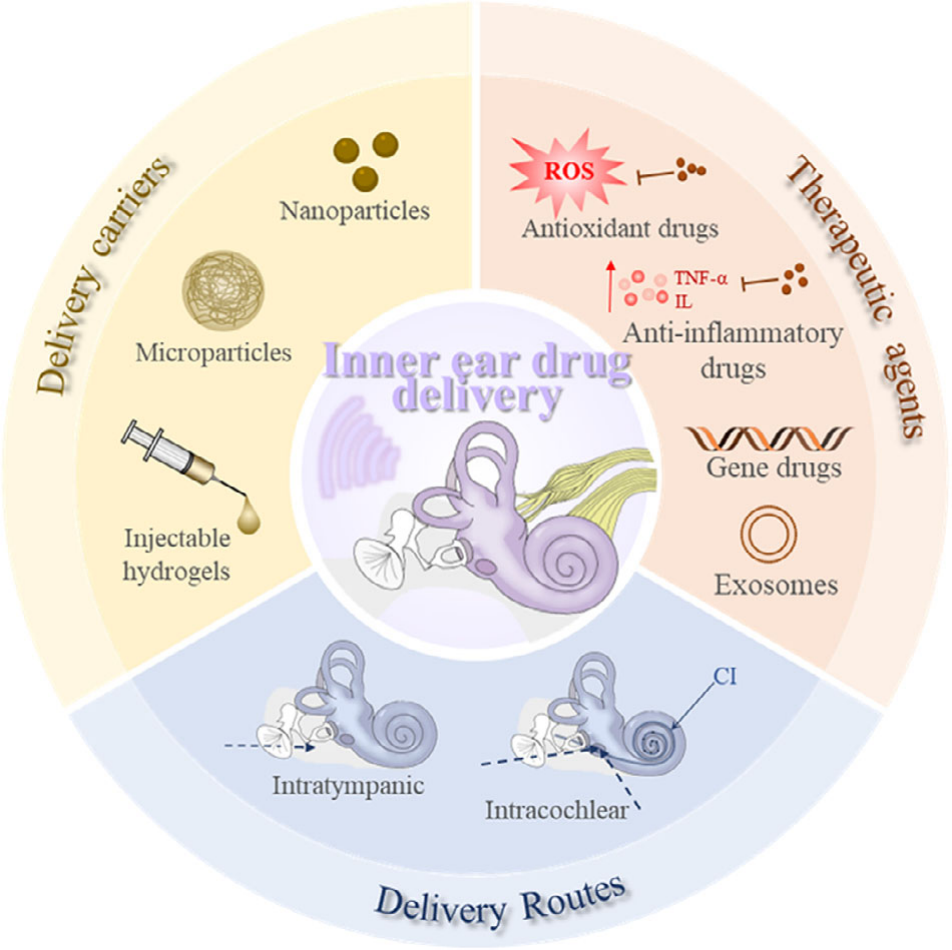
Drug delivery systems for sensorineural hearing loss.

Herein, we reviewed the development of drug delivery systems for the treatment of sensorineural hearing loss (**Figure** **1**). We commenced by introducing the physiological structure of the inner ear and highlighting the intricacy of its anatomy and the challenges associated with drug administration. We then classified the drugs currently used in these delivery systems. Subsequently, we introduce current drug delivery technologies and summarized their limitations and shortcomings. We then provide a detailed account of the latest carriers used in inner ear drug delivery systems. Finally, we outline the future development trajectory and potential breakthrough points for inner ear drug delivery systems. We hope that this review will provide theoretical support and inspiration for the development of hearing loss treatment technologies and attract more interest from researchers in related fields.

## 2 The Barriers to Inner Ear Drug Delivery

### 2.1 The Anatomical Structure of the Inner Ear

**Figure 2:**
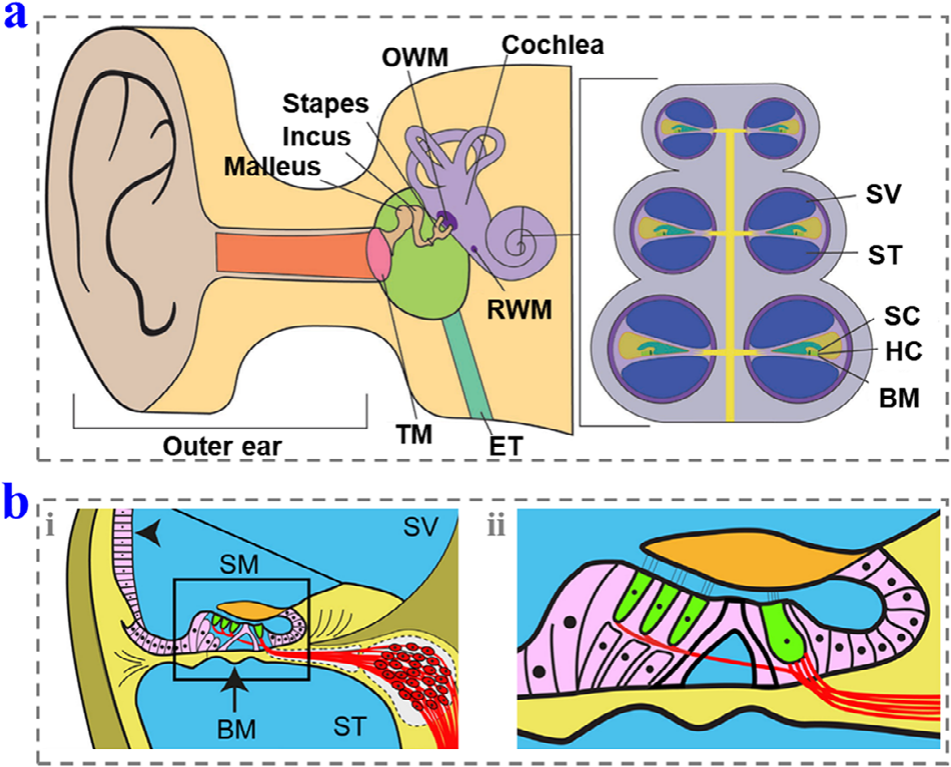
a) The anatomical structure of the i) human ear, ii) cochlea, and iii) Corti. Reproduced with permission.^[^ ^22^ ^]^ Copyright 2022, Wiley‐VCH. b) The anatomical structure of BLB. Reproduced with permission.^[^ ^23^ ^]^ Copyright 2019, Elsevier. OWM: oval window membrane; RWM: round window membrane; ET: Eustachian tube; TM: tympanic membrane; SV: scala vestibuli; ST: scala tympani; SC: stereocilia; HC: hair cell; BM: basilar membrane; SM: scala media.

The inner ear, also known as the labyrinth, is a complex and delicate structure consisting of the vestibular system and the cochlea. It is the primary organ of proprioceptive sensation and auditory. The blood labyrinth barrier (BLB), the round window membrane (RWM), and the oval window membrane (OWM) separate it from the circulatory system and the middle ear (**Figure** **2**a).^[^ ^22^ ^]^ The cochlea is the primary structure for hearing in the inner ear, and it has a spiral shape that resembles a tiny snail shell. The main body of the cochlea is the cochlear duct, which is a hollow bony tube filled with lymphatic fluid, and narrows from the bottom to the top and is divided into the basal, middle, and apical turns. The cochlear duct has a basilar membrane, which is covered with inner and outer hair cells (IHCs and OHCs). When the sound wave transmits to the inner ear, it causes fluctuations in the lymphatic fluid and the subsequent displacement of the HCs. This displacement results in the electrochemical signal changes within the HCs, which further induces the release of neurotransmitters and the forming of electrical signals. These electrical signals are then transmitted to the auditory center in the cerebral cortex, where they are identified as sound. Furthermore, the cochlea contains additional supporting structures, including the capping membrane and the spiral ligament, which collectively maintain its structural integrity and functionality.

### 2.2 BLB

The BLB is a complex structure that acts as a physicochemical barrier between the body's circulation system and the cochlea (Figure 2b).^[^ ^23^ ^]^ It is similar in function to the blood–brain barrier and is composed primarily of the blood–external lymphatic barrier, the blood–endolymphatic barrier, and the endolymphatic–external lymphatic barrier.^[^ ^24^ ^]^ The blood–endolymphatic barrier, which is situated in the stria vascularis of the cochlea, represents the core structure of BLB functionality and is primarily constituted of marginal cells, intermediate cells, and basal cells.^[^ ^25^ ^]^ Both marginal and basal cells are connected by tight junctions, while intermediate cells are interspersed with large cell protrusions and folds. The intermediate cell layer is characterized by the presence of a distinct capillary network comprising endothelial cells, pericytes, perivascular‐resident‐macrophage‐like melanocytes (PVM/Ms), and the basement membrane.^[^ ^26^ ^]^ Endothelial cells represent the primary component of the barrier, exhibiting a virtually impermeable cell membrane and markedly low permeability, thereby enabling precise regulation of mass transfer. Pericytes and PVM/Ms, on the other hand, play a pivotal role in modulating barrier permeability and stability through matrix deposition and the release of activation signals. Meanwhile, the basement membrane provides structural support for the barrier.

The BLB permits the passage of essential nutrients and metabolites that facilitate the normal metabolism and function of inner ear cells. Meanwhile, it protects the inner ear from toxic substances and pathogens present in the blood by preventing the passage of the majority of microorganisms, toxins, macromolecules, and compounds from the blood into the lymphatic fluid of the inner ear, thereby maintaining a relatively stable environment within the inner ear. This highly selective permeability ensures the maintenance of a clean and stable inner ear environment. However, it limits the effectiveness of treatments for inner ear disorders. The drugs with enough lipophilicity can permeate the BLB easily, while these drugs are estimated to be less than 1%.^[^ ^24^ ^]^ Although BLB is similar to blood–brain barrier (BBB), and the small molecule drugs that can across the BBB have been studied for featuring with fewer than eight hydrogen bonds and a molecular weight of smaller than 400 Da, the similar study in BLB is still lacking.^[^ ^27^ ^]^ The usage of glycerin can open the BLB, but its efficiency should be discussed in detail.^[^ ^28^ ^]^ Although the assistance of ultrasound and microbubbles could promote the drug loaded by the nanoparticles (NPs, hydrodynamic size was about 300 nm) across BLB, more clear and solid evidence is still lacking.^[^ ^29^ ^]^ In fact, there are many parameters that affect the permeating efficiency of NPs, including the size, shape, and modification. It has been proven that in the intestinal uptake and transport study, the uptake of NPs of 50 nm was quicker than the larger NPs, and the transport across monolayer capability of the NPs of 50 nm (25% for 5 h) was similar to that of 200 nm but much higher than 500 (15%) and 1000 (8%) nm.^[^ ^30^ ^]^ Besides, the shape of the NPs also affects their transport efficiency. Rod‐ (15% for 5 h) and disc‐ (18%) like NPs showed higher transport efficiency than the spherical (11%) NPs. Moreover, the surface modification of NPs also increased the transport efficiency. Nevertheless, the solid studies in permeating BLB are still unexplored.

### 2.3 RWM and OWM

The RWM is a crucial structure of the inner ear, situated at the base of the cochlea (the round window niche). It is flexible and permeable and works in conjunction with the OWM to facilitate the movement of fluid within the basilar membrane by oscillating in opposing phases, thereby ensuring that the HCs are adequately stimulated and finally cause sound perception. The OWM, also referred to as the vestibular window, constitutes a vital component of the inner ear, situated between the middle ear and the inner ear. The OWM serves as a conduit for sound waves to enter the inner ear and converts the mechanical energy of sound waves into fluctuations in the lymphatic fluid within the inner ear, which further stimulates the production of nerve impulses.

In the drug delivery systems, the RWM and OWM serve crucial functions as barriers as well as channels.^[^ ^31^ ^]^ Since the existence of BLB, the systemic administration suffers low drug concentrations in reaching the inner ear and rapid metabolization. By contrast, RWM and OWM, which are semipermeable membranes between the middle ear and the inner ear, allow the passage of drugs and facilitate an increased drug concentration through local administration techniques. This method is promising to significantly enhance drug bioavailability and reduce side effects, which is crucial for the management of inner ear disorders. However, the complex structure of the RWM and OWM can affect the drug delivery efficiency, which closely relates to drugs’ or drug carriers’ physicochemical properties, including their size, shape, modification, and so on. Generally, the drug carriers are in nanoscale (smaller than 1000 nm), and the smaller size showed better permeating efficiency, while the carriers with a diameter of 5 ± 0.5 µm cannot across the RWM.^[^ ^32^ ^]^ Furthermore, the rapid clearance of the Eustachian tube can significantly reduce the drug concentration within the tympanic chamber, which in turn reduces the drug delivery efficiency. Therefore, drug delivery systems must prioritize the penetration of drugs into the RWM and OWM, as well as their retention within the tympanic chamber.

## 3 Therapeutic Agents for Sensorineural Hearing Loss

Although there are currently no effective drugs that can be widely used to treat hearing loss, many preclinical and clinical studies have been conducted, and a variety of agents have shown promising in applications. According to the effects of the drugs, they can be divided into antioxidant drugs, anti‐inflammatory drugs, gene drugs, and so on. Besides, although exosomes (Exos) are hard to be defined as drugs due to their complex components and biological processes, they can indeed play therapeutic effects via many possible pathways, and have been widely explored as therapeutic agents for many diseases.^[^ ^33^ ^]^ Therefore, we also discuss their roles in hearing loss treatment.

### 3.1 Antioxidant Drugs

Oxidative stress, a state in which the production of ROS in the body is too high and exceeds the clearance capacity of the antioxidant system and results in cell damage, is considered to be one of the main mechanisms causing sensorineural hearing loss.^[^ ^34^ ^]^ Excess ROS weakens the antioxidant capabilities of the cells, leading to cytochrome C release, which activates the caspase pathways and triggers apoptotic cell death, and further activates caspase‐3 and caspase‐9 that are closely related to deoxyribonuclease activity and cause DNA damage.^[^ ^35^ ^]^ Mitochondria are the main sites for ROS production, and thus, they are attacked by high level ROS first. The high level ROS results in the damage of mitochondrial DNA, affecting the function and energy production of the mitochondria, leading to the damage of cells. ROS also cause lipid peroxidation and protein oxidation within the cell. Besides, ROS may activate the apoptotic pathways of the stria vascularis or the SGNs via nuclear factor kappa B (NF‐κB).^[^ ^36^ ^]^ Therefore, the usage of antioxidant drugs is a feasible strategy for sensorineural hearing loss (**Table** **1**).

**Table 1:** The therapeutic agents for inner ear protection. Abbreviations. ROS: reactive oxygen species; s.c.: subcutaneous injection; NAC: *N*‐acetylcysteine; EbSe: ebselen; GPx: glutathione peroxidase; TTS: temporary threshold shift; AHL: age‐related hearing loss; ALA: α‐lipoic acid; STS: sodium thiosulfate (Na_2_S_2_O_3_); OHCs: outer hair cells; IHCs: inner hair cells; PNS: panax notoginseng saponins; TSIIA: tanshinone IIA; SOD: superoxide dismutase; HCs: hair cells; DEX: dexamethasone; MP: methylprednisolone; SHL: sudden hearing loss; TNF‐α: tumor necrosis factor‐α; KLH: keyhole limpet hemocyanin; PTA: pure tone average; IL‐1: interleukin 1; MWS: Muckle–Wells syndrome; *VGLUT3*: vesicular glutamate transporter‐3; AAV: adeno‐associated virus; vg: vector genomes; KO: knockout; ABR: auditory brainstem response; gc: gene copies; PSCC: posterior semicircular canal; CKO: conditional knockout; CMV: cytomegalovirus; *TMC1*: transmembrane channel‐like 1; WPRE: woodchuck hepatitis virus (WHP) post‐transcriptional regulatory element; *OTOF*: otoferlin; *USH1C*: usher syndrome 1C; ASO: antisense oligonucleotide; BBN: broad band noise; SCCF: semicircular canal fenestration; sgRNA: single guide RNA; MYO6: myosin VI; SM: scala media; Kcnq4: potassium voltage‐gated channel subfamily Q member 4; NHEJ: nonhomologous end‐joining; m‐3j‐gRNA1: mutant‐av3j‐guide RNA; Pcdh15: protocadherin related 15; JNK: c‐Jun N‐terminal kinase; CDK2: cyclin‐dependent kinase 2; BDNF: brain‐derived neurotrophic factor; TRKB: tropomyosin receptor kinase B; NIHL: noise‐induced hearing loss; NT‐3: neurotrophin‐3; PSD: postsynaptic densities; JWH015: (2‐methyl‐1‐propyl‐1*H*‐indol‐3‐yl)‐1‐naphthalenylmethanone; CB2R: cannabinoid receptor 2; RGS17: regulators of G protein signaling 17.

Classification	Drugs	Mechanisms	Dosage	Administration routes	Objects	Outcome/efficiency rate	Ref.
Antioxidant drugs	Glutathione	Reacts with ROS	1.2 g kg^−1^ per day	Oral	Guinea pigs treated with gentamicin sulfate	43 dB lower ABR thresholds at 3 kHz, 27 dB lower at 8 kHz, and 21 dB lower at 18 kHz	[81]
	NAC	Prodrug of glutathione	20 mm	Mixing with medium	Cochlear organotypic culture treated with 50 µm cisplatin	Completely protected HCs	[82]
	EbSe	GPx activity	10 mg kg^−1^, 1 h before noise exposure	Oral	Guinea pigs treated with 115 dB 4 kHz octave band noise for 3 h	Eliminated noise‐induced TTS	[83]
	Vitamin C	Reacts with ROS	4.44 mg per day	Oral	Fischer 344 rats for AHL	Showed a statistically significant lower threshold compared with the placebo group at all frequencies except 3 kHz	[84]
	Vitamin E	Eliminating radicals of lipid peroxide and ending lipid peroxidation chain reactions	4 g kg^−1^	Intratympanic administration	Rats treated with 20 mg kg^−1^ cisplatin 30 min after vitamin E injection	4 dB lower ABR thresholds at 4 kHz, 1.2 dB lower at 8 kHz, 60.3 dB lower at 12 kHz, and 58.6 dB lower at 16 kHz	[85]
	ALA	Protecting mitochondrial functions	150 mg kg^−1^ diet	Oral	B6 mice from 4 to 15 months	About 35 dB lower ABR thresholds at 8 and 16 kHz, and 60 dB lower at 32 kHz	[86]
	STS	Scavenging ROS and chelating platinum drugs	10 mm	Osmotic minipump perfusion	Guinea pigs treated with a daily intraperitoneal injection of cisplatin (2 mg kg^−1^ per day) for 5 consecutive days	Protected more than 92.8% of OHCs and IHCs destined to die	[46]
	PNS and TSIIA	Downregulating malondialdehyde and lipid peroxide and elevating the activity of SOD	100 µL, 3 mg mL^−1^	Tympanic injection	Guinea pigs treated with 12 mg kg^−1^ cisplatin or 400 mg kg^−1^ kanamycin	Almost completely preserve the HCs and remain the hearing threshold shift within normal limits	[48]
Anti‐inflammatory drugs	DEX	Inhibiting the production of inflammatory factors	Cubosomes, particle size<220 nm, 4 mg kg^−1^	Intratympanic injection	Male Wistar rats treated with 10 mg kg^−1^ cisplatin 24 h before or 30 min after DEX injection	37.91 dB lower ABR thresholds at 1.5 kHz, 22.54 dB lower at 8 kHz at day 14 for protection, and 49.92 dB lower ABR thresholds at 1.5 kHz, 48.76 dB lower at 8 kHz at day 14 for therapy	[87]
	MP		1 mg kg^−1^, maximum 48 mg for oral, and 1 mL, 40 mg mL^−1^ for injection	Oral/retroauricular injection/intratympanic injection	Patients with SHL or Meniere's disease	27.44 ± 12.71 dB lower ABR thresholds for oral, 29.13 ± 10.66 dB lower for retroauricular injection, and 28.47 ± 10.20 dB lower for intratympanic injection	[88]
	Etanercept	Blocking TNF‐α activity	5 mg for systemic administration or 5.0 µg h^−1^ for 7 days for local infusion	Systemic administration or local infusion	Guinea pigs treated with KLH	34 dB lower ABR thresholds for systemic administration, and 41 dB lower for local infusion	[55]
	Infliximab		0.3 mL per week, Remicade, schering plough	Intratympanic administration	Patients presenting with autoimmune SHL	22.6 ± 15.7 dB lower ABR thresholds for PTA	[89]
	Anakinra	Competitively binding to the IL‐1 receptor	Not mentioned	Not mentioned	Patient presenting with MWS	15–30 dB improvement in the 250–4000 Hz frequency range in each ear	[90]
Gene replacement	AAV1–*VGLUT3*	Replacing the *VGLUT3* gene	0.6 or 1.0 µL, 2.3 × 10^13^ vg mL^−1^ for RWM injection; 0.6 µL, 2.3 × 10^13^ vg mL^−1^ for cochleostomy	RWM injection and cochleostomy	*VGLUT3* KO mice	ABR thresholds normalize, along with partial rescue of the startle response	[59]
	AAV8–*VGLUT3*		0.5 and 1 µL at a rate of 169 nL min^−1^ for neonatal and adult mice, 3.87 × 10^13^ gc mL^−1^	Cochleostomy for neonatal and PSCC perforation for adult mice	*VGLUT3* KO mice	Auditory function was successfully restored, and the hearing threshold remained stable for at least 12 weeks	[91]
	AAV1–*GJB2*	Replacing the *GJB2* gene	0.05 and 0.02 µL min^−1^ in adult and neonatal mice for 10 min, with a concentration of 8.6 × 10^11^ viral particles	RWM injection	*GJB2* CKO mice	About 15 dB lower ABR thresholds at 12 kHz, 23 dB lower at 24 kHz for neonatal mice; no significant change in the adult mice	[92]
	AAV2/9‐PHP.B–CMV–*TMC1*–WPRE	Replacing the *TMC1* gene	1 µL, 5.64 × 10^10^ gc	Utricle injection	*TMC1* KO mice	About 60 dB lower ABR thresholds at 11.3 kHz	[93]
	AAV2/Anc80L65–CMV–*TMC1*–WPRE		1 µL, 1.4 × 10^11^ gc	RWM injection	*TMC1* KO mice	ABR thresholds of the treated mice were ranged from 35 to 90 dB and corresponded to best frequencies of 8–16 kHz, while those of the untreated mice were higher than 110 dB at all the frequencies	[94]
	Dual AAV1–*hOTOF*	Replacing the *OTOF* gene	One received a dose of 9 × 10^11^ vg, 30 µL, and five received 1.5 × 10^12^ vg, 50 µL	RWM injection with stapes fenestration	Children (aged 1–18 years) with severe‐to‐complete hearing loss and confirmed mutations in both alleles of *OTOF*	About 40–57 dB lower ABR thresholds at 0.5–4.0 kHz	[12b]
Gene suppression	ASOs	Correcting defective pre‐mRNA splicing of transcripts from the *USH1C* gene with the c.216G>A mutation	300 mg kg^−1^	Intraperitoneal injection	Neonatal mice with *USH1C* c.216G>A mutation	Hear at 6 months of age to BBN and 8 and 16 kHz	[95]
	AAV2/9 containing microRNA	RNAi‐mediated gene silencing	1 µL, 3.3 × 10^10^ vg	RWM injection + SCCF	*TMC1* Bth mice	25–30 dB lower in click‐evoked ABR thresholds	[96]
Gene editing	Cas9–*TMC1*–mut3 sgRNA complex	Targeting the *TMC1* Bth allele	0.3 µL for the base, middle, and apex, respectively, 69 nL min^−1^ for neonatal mice; 1 µL, 169 nL min^−1^ for adult mice	Cochleostomy for neonatal mice and PSCC for adult mice	*TMC1* Bth mice	Average 15 dB lower ABR thresholds	[97]
	SaCas9–KKH–Myo6‐g2 packaged in AAV–PHP.eB	Disruption of the Myo6^C442Y^ allele	500 nL, 3.73 × 10^13^ gc mL^−1^, 169 nL min^−1^	SM injection	Myo6 p.C442Y mice	About 22 dB lower ABR thresholds	[63]
	AAV2/Anc80L65 packaged plasmids of split Cas9 and gRNA	Disrupting the dominant‐negative allele in Kcnq4	1 µL, 40 nL min^−1^, 1 × 10^9^ gc µL^−1^	RWM, SM, PSCC, and utricle injection	Kcnq4 c.827G>C mice	10–20 dB lower ABR thresholds	[98]
	AAV2/9 packed the m‐3j‐gRNA1 containing U6‐gRNA scaffold	NHEJ‐mediated nonrandom editing profiles compensate the frameshift mutation	600 nL, 1.5 × 10^14^ vg mL^−1^, 250 nL min^−1^	SM injection	Pcdh15^av‐3j^ mice	The best‐performing mice showed visible click ABR thresholds at 75 dB	[99]
	Dual AAV encoding AID–BE3.9max + sgRNA1	Corrected the *TMC1* point mutation back to the wild‐type base	1 µL, 6.2 × 10^9^ vg	Inner ear injection	*TMC1*‐Baringo mice	At least 50 dB lower ABR thresholds compared with untreated *TMC1*‐Baringo mice	[64]
Exos	Exos	Interacting with TLR4 on HCs, regulating autophagy, etc.	30–150 nm, 20 µg in 10 µL PBS	Round window niche injection	Mice treated with neomycin once per day for five consecutive days at a dose of 200 mg kg^−1^	About 10 dB lower ABR thresholds	[66a]
Apoptosis regulation	D‐JNKI‐1	JNK inhibitor	86.7 ± 5.30 nm, 0.2 µL	Round window niche administration	Mice treated with broadband white noise at 115–120 dB for frequencies between 6.3 and 20 kHz for 4 h	About 15–35 dB lower ABR thresholds	[71b]
	Pifithrin‐α	p53 inhibitor	20, 40, 60, or 100 µm	Mixing with medium	Cochlear organotypic culture treated with 1, 5, or 10 µg mL^−1^ cisplatin for 48 h	Protected almost all the HCs when pifithrin‐α was 100 µm	[74a]
	Kenpaullone	CDK2 inhibitor	5 µm	Mixing with medium	hiPSC‐derived otic organoids 100 µm cisplatin	Prevented the upregulation of cleaved caspase‐3, and protected against cisplatin‐induced neural damage initially	[100]
Neuron protection	BDNF	Activating the TRKB signaling pathway	50 µg mL^−1^, 10 µL per ear	Intratympanic injection	Mice treated with single noise exposure (115 dB, 4 h)	Completely restores NIHL	[101]
	NT‐3	Increasing axon growth and synaptogenesis	3.6 nm	Mixing with medium	Rat cochlear explant treated with glutamate receptor agonists 0.5 mm NMDA and 0.5 mm kainic acid for 2 h	The PSD was about threefold of that of the untreated groups	[102]
	JWH‐015	CB2R agonist	0.815 µg per ear	Transtympanic administration prior to cisplatin	Rats treated with 11 mg kg^−1^ cisplatin by intraperitoneal injections	Reduced RGS17 expression from 4.5 ± 1.0‐fold to 1.1 ± 0.2	[103]
	Caroverine	Antagonist of glutamate receptors	160 mg per 8 mL in 100 mL physiological saline, 2 mL min^−1^.	Intravenous administration	Patients suffering from cochlear synaptic tinnitus	Significant fall in tinnitus grading and matching immediately, which raised at 3 and 6 months follow‐up	[104]

Glutathione is the most important nonenzymatic antioxidant in the body.^[^ ^37^ ^]^ There are two main forms of glutathione in vivo: reduced (GSH) and oxidized (GSSG). GSH has a strong antioxidant capacity and can directly eliminate ROS in the body, relieve oxidation reactions, and protect cells from oxidative damage. It can also enhance the antioxidant capacity of other antioxidants. Besides, GSH has a self‐recycling mechanism in vivo: after being oxidized into GSSG, it can be transformed back into GSH by glutathione reductase and coenzyme, so as to realize the continuous antioxidant effect.

*N*‐acetylcysteine (NAC) is a derivative of the amino acid cysteine, acts as a precursor for glutathione, and improves the intracellular glutathione levels.^[^ ^38^ ^]^ It also plays other important roles in the body, including scavenging free radicals, protecting mitochondria, and inhibiting glutamate excitotoxicity and lipid peroxidation. Its protective effects on the cochlea have been noted, particularly for drug‐induced hearing loss and noise‐induced hearing loss (NIHL), which is realized by scavenging ROS directly and may also enhance the detoxification of toxic metabolites in the inner ear.

**Figure 3:**
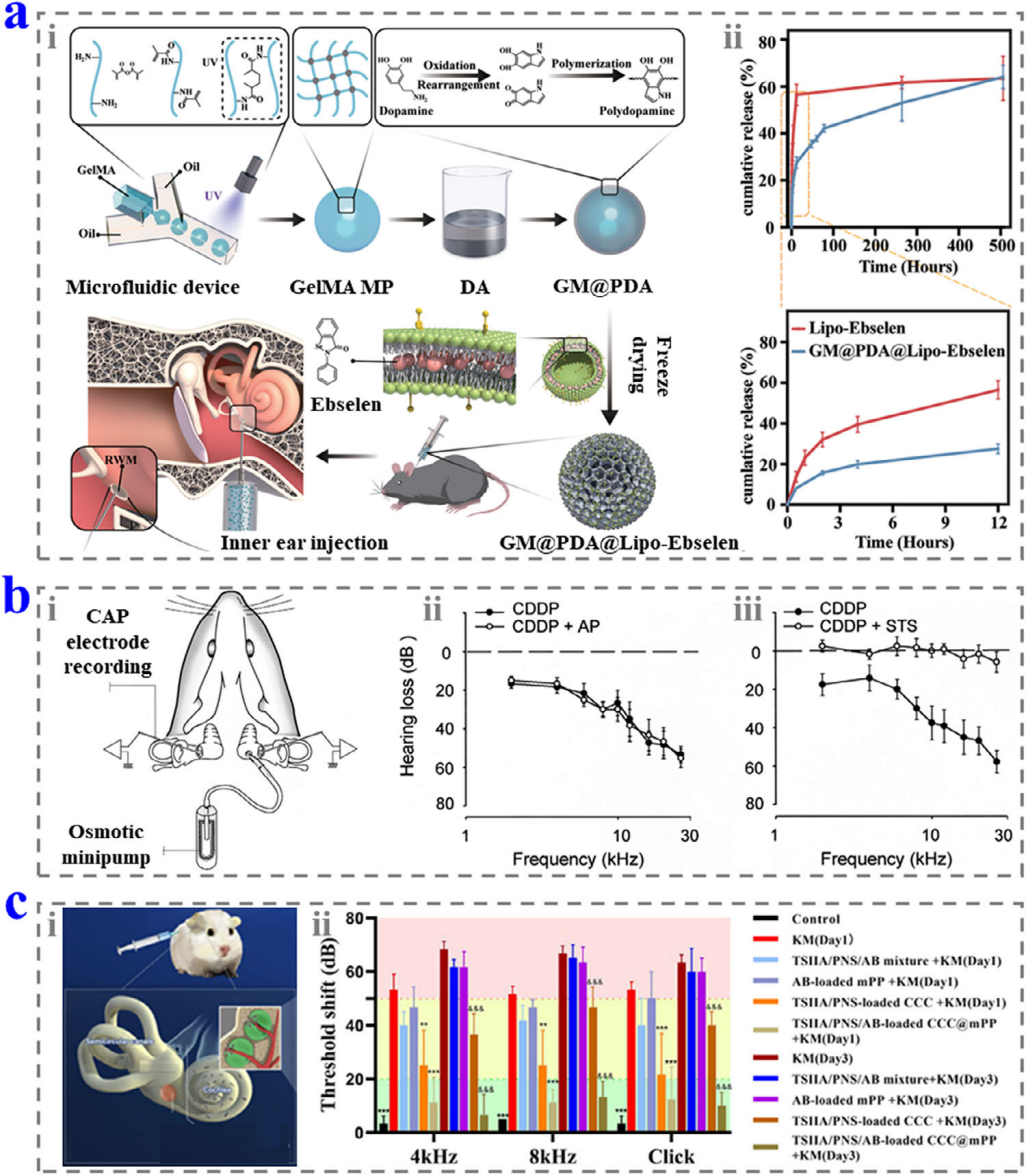
a) i) Schematic diagrams of the fabrication and application of the EnSe‐loaded microcarriers. ii) The drug release properties of the microcarriers. Reproduced with permission.^[^ ^42^ ^]^ Copyright 2022, Wiley‐VCH. b) i) Schematic diagrams of the STS delivery device. ii, iii) The hearing loss of the mice treated without STS (ii) and with STS (iii). Reproduced with permission.^[^ ^46^ ^]^ Copyright 2003, Elsevier. c) i) Schematic diagram of the application of CCC@mPP NPs in hearing loss protection. ii) The auditory brainstem response (ABR) tests of the mice in the different groups. Reproduced with permission.^[^ ^48^ ^]^ Copyright 2023, American Chemical Society.

Ebselen (EbSe) is a synthetic, lipid‐soluble, selenium‐containing organic small molecule with glutathione peroxidase (GPx) activity.^[^ ^39^ ^]^ It inhibits processes such as lipid peroxidation by scavenging intracellular ROS, thereby reducing the oxidative stress damage to inner ear cells.^[^ ^40^ ^]^ It reduces OHC loss and stria vascularis damage in a variety of animal models, which is critical for maintaining hearing function.^[^ ^41^ ^]^ In addition, selenium in the molecule is difficult to release in the body, resulting in low toxicity and good biosafety. Chen et al. employed EbSe as the drug to treat NIHL via encapsulating it into the hydrogel microcarrier generated from microfluidics and coated by polydopamine (PDA).^[^ ^42^ ^]^ The microcarriers could be delivered to the middle ear by injection and adhered to RWM tightly (**Figure** **3**a). When the mice were treated by the microcarriers, they realized a 93 ± 2% survival rate of OHCs. By contrast, that of the mice without treatment was only 48 ± 6%, indicating a good therapeutic effect.

Vitamin C and vitamin E are two important water‐soluble and lipophilic‐soluble antioxidants, respectively.^[^ ^43^ ^]^ Due to their powerful antioxidant properties, they protect the HCs in the cochlea from oxidative stress (dose‐dependent, and the quartile points are 44.623, 107, and 213.5 mg per day for vitamin C and 0.944, 6.444, and 22.2 mg per day for vitamin E, respectively).^[^ ^43a^ ^]^ In addition, they improve microcirculation and increase the blood supply to the cochlea, providing more nutrients and oxygen. In addition, the lipophilic‐soluble nature of vitamin E makes it particularly suitable for protecting the membrane of HCs.

α‐lipoic acid (ALA). is a powerful antioxidant that effectively scavenges ROS in the body, preventing them from causing oxidative damage to cochlear cells and tissues, thereby alleviating hearing loss.^[^ ^44^ ^]^ ALA also protects mitochondrial function by reducing the accumulation of ROS (100 mg kg^−1^ prior damage).^[^ ^44a^ ^]^ Mitochondria are the intracellular “energy factories” that are essential for normal cochlear cell metabolism and hearing function. Impaired mitochondrial function is one of the major pathological processes in cisplatin‐induced ototoxicity. The protective effect of ALA can prevent mitochondria deterioration, thereby reducing hearing loss.

Sodium thiosulfate (Na_2_S_2_O_3_, STS) is a thiol‐containing antioxidant and has shown good potential and application prospects in reducing the sensorineural hearing loss caused by cisplatin and other chemotherapy drugs.^[^ ^45^ ^]^ It can react with ROS to reduce damage to HCs. Moreover, it has a good affinity with platinum drugs, indicating that it is capable of binding platinum‐based drugs such as cisplatin to form stable complexes in the inner ear to prevent cell toxicity (1600 mg kg^−1^, 30 min before injection).^[^ ^45a^ ^]^ These features suggest that it is promising for the hearing loss treatment of the inner ear, while it may affect the therapeutic effects of the antitumor drugs. Wang et al. designed a device to deliver STS into the cochlea directly to avoid the adverse effect (Figure 3b).^[^ ^46^ ^]^ They opened a small hole close to the RWM and inserted a glass perfusion pipette into the cochlea, which was further fixed by dental cement and connected to a pump. This device could efficiently deliver STS into the inner ear and protect the HCs and stria vascularis from cisplatin‐induced damage, thus protecting the hearing.

Many plant extracts have also been found to have powerful antioxidant properties, and thus, they also show the potential to protect inner ear from hearing loss.^[^ ^47^ ^]^ For example, Chen and co‐workers employed panax notoginseng saponins (PNS) and tanshinone IIA (TSIIA), two kinds of Chinese medicine compounds, together for cisplatin‐induced hearing loss treatment.^[^ ^48^ ^]^ In the work, the drugs were loaded by the composite NPs (CCC@mPP NPs) which could enter the inner ear. The CCC@mPP NPs could protect almost all the HCs and maintain the hearing threshold from the treatment of aminoglycoside and cisplatin, indicating they are reliable for sensorineural hearing loss therapy (Figure 3c).

Antioxidants are able to target the oxidative stress mechanisms directly, thus have a significant advantage in preventing and improving hearing loss. Despite their excellent performance in animal studies, their use in clinical therapy is still limited due to a lack of research into the accurate timing, dosage, and administration in treating sensorineural hearing loss. In addition, the long‐term safety and efficacy of antioxidant drugs need to be further verified in the clinic. Moreover, although they are promising to prevent hearing loss caused by ototoxic drugs, they may also interfere with the treatment effect.

### 3.2 Anti‐Inflammatory Drugs

Noise, ototoxic drugs, viral infections, and other factors can activate the inflammatory response in the inner ear. The immune cells release many inflammatory factors, including tumor necrosis factor‐α (TNF‐α) and interleukin (IL). These inflammatory factors activate a variety of pathways, such as NF‐κB, caspase, and mitogen‐activated protein kinase/c‐Jun N‐terminal kinase(JNK), resulted in the apoptotic in the inner ear. Thus, inflammation is another important reason for the occurrence of sensorineural hearing loss, and the application of anti‐inflammatory drugs that can regulate the inflammatory response is an important means of treatment for sensorineural hearing loss.^[^ ^49^ ^]^ The main anti‐inflammatory drugs applied in treating sensorineural hearing loss include hormonal drugs and biologics (Table 1).

**Figure 4:**
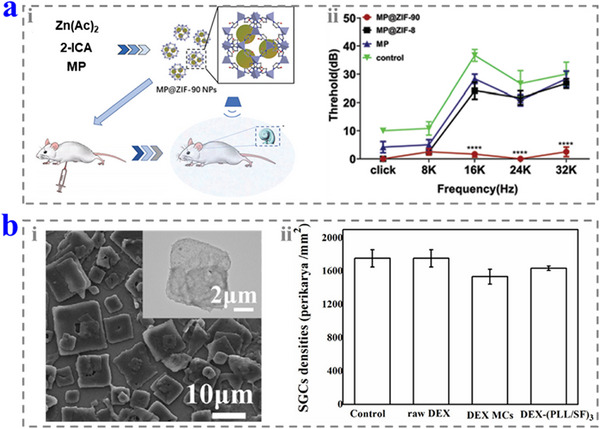
a) i) Schematic diagrams of the fabrication and application of the MP‐loaded ZIF‐90. ii) The protective properties of the MP‐loaded ZIF‐90. Reproduced with permission.^[^ ^51^ ^]^ Copyright 2020, Royal Society of Chemistry. b) i) Scanning electron microscopy (SEM) image of the DEX─(PLL/SF)_3_. ii) The densities of spiral ganglion cells in the different groups. Reproduced with permission.^[^ ^52^ ^]^ Copyright 2020, Elsevier.

Glucocorticoid drugs are the main drugs used in the treatment of sudden deafness, and they have powerful anti‐inflammatory and immunosuppressive effects. It relieves the inflammatory response of the inner ear by inhibiting the production of inflammatory factors in the cochlea and thus, protects the auditory nerve and HCs. The commonly used hormonal drugs include methylprednisolone (MP) and dexamethasone (DEX).^[^ ^50^ ^]^ MP has better penetration of the RWM, whereas DEX can be absorbed quickly by the stria vascularis and surrounding tissues and has stronger anti‐inflammatory efficacy. These drugs are often modified with polar groups to increase water solubility and thus facilitate intravenous administration. However, when they are administered locally, the modification may instead reduce their permeability in the RWM. Xu et al. employed the zeolitic imidazolate framework‐90 (ZIF‐90) NPs to load MP and deliver the NPs by intraperitoneal injection for NIHL treatment (180 mg kg^−1^).^[^ ^51^ ^]^ Compared with the free drugs or drugs loaded by ZIF‐8, the presented NPs showed the best therapeutic effect (**Figure** **4**a). Li et al. designed a DEX microcrystal (DEX MC) using precipitation technology and coated it with silk via layer‐by‐layer assembly to form a DEX─(PLL/silk fibroin (SF))_3_ (2 mg).^[^ ^52^ ^]^ The coating promoted the uniform distribution of the MCs on the RWM while maintaining a high concentration of DEX in perilymph without significant side effects (Figure 4b).

Biologics are a novel type of anti‐inflammatory drugs used to treat sensorineural hearing loss.^[^ ^53^ ^]^ They are target‐specific and easily absorbed by the body and exert anti‐inflammatory effects by inhibiting specific inflammatory factors.^[^ ^54^ ^]^ Common biologics include TNF inhibitors and IL inhibitors. For example, etanercept blocks the activation of the TNF‐α pathway and inhibits the inflammatory cascade by preventing the binding of TNF‐α to its receptor (2.5 mg systematical administration before modeling + 2.5 mg intraperitoneal dose 3 days later, or 5.0 µg h^−1^ for 7 days by long‐term infusion into the scala tympani using an osmotic pump).^[^ ^55^ ^]^ Gevokizumab binds to IL‐1β and blocks the induced inflammatory response to protect against hearing loss.^[^ ^56^ ^]^ Anakinra can competitively bind to the IL‐1 receptor and block the binding, thus effectively reducing hearing loss.^[^ ^57^ ^]^

Anti‐inflammatory drugs can protect inner ear by rapidly improving the inner ear microenvironment and reducing inflammatory damage. However, the use of large amounts of hormonal drugs in treatment can lead to serious side effects. By contrast, biologics have relatively fewer side effects, while they are expensive because of the high technical requirements and strict conditions of storage and transport.

### 3.3 Gene Drugs

Genetic reasons cause about half of all hearing loss. They can not only cause deafness directly, but also increase the risk of deafness caused by noise, drugs, and aging. Therefore, gene therapy is a promising technology for hearing loss treatment. Gene therapy strategies include gene replacement, gene suppression, and gene editing, and they can be used according to the pathogenic mechanism.^[^ ^58^ ^]^

**Figure 5:**
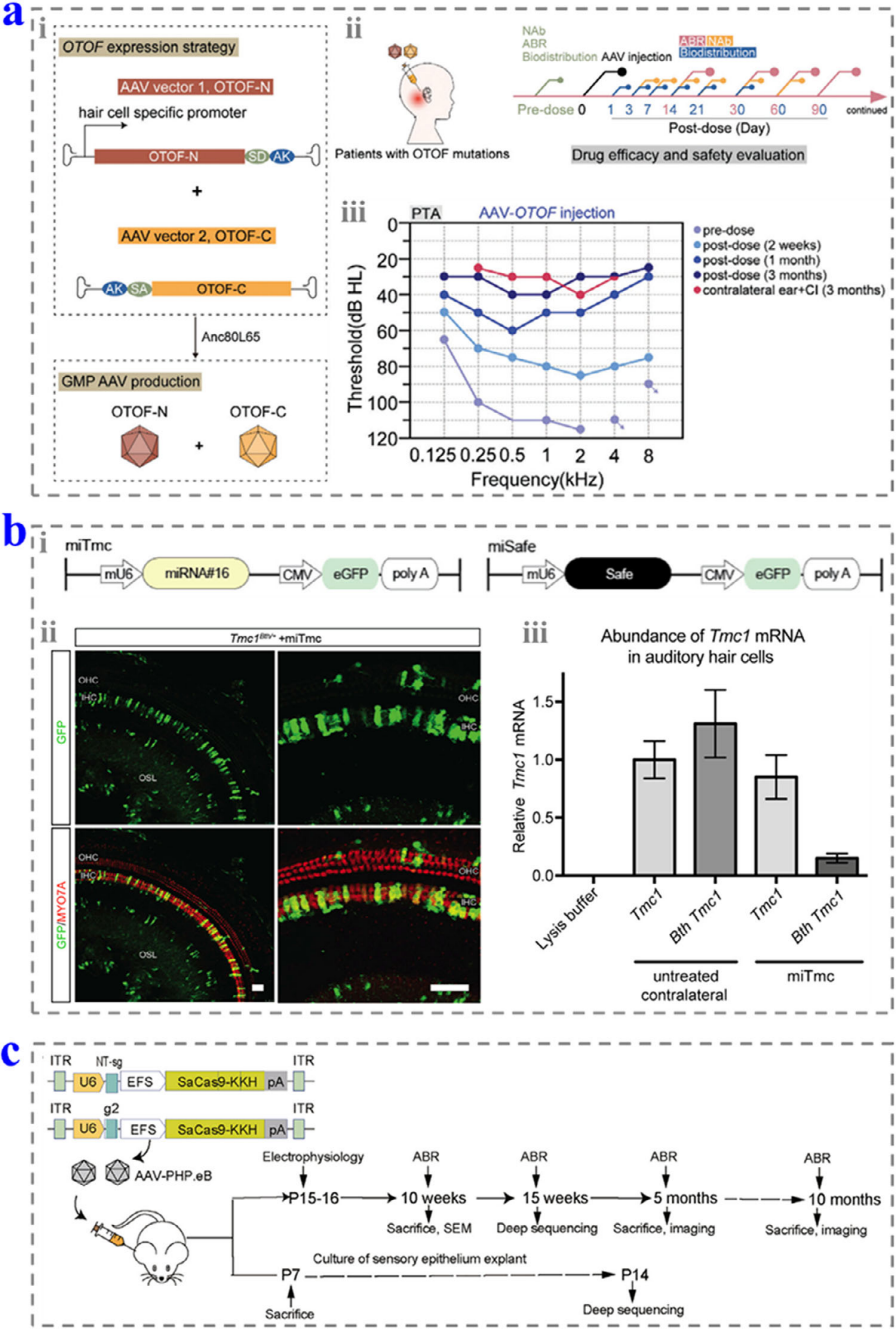
a) i) Schematic diagrams of constructing *OTOF*‐packaged dual AAV vectors. ii) The therapeutic strategy of AAV–*OTOF* in patients with *OTOF* mutations. iii) The pure tone average (PTA) results of the patient after treatment. Reproduced with permission.^[^ ^12c^ ^]^ Copyright 2024, Wiley‐VCH. b) i) The schematic diagrams of constructing the miRNA expression cassette for *TMC1*‐mutation‐caused deafness. ii) The eGFP localization in the treated Corti. iii) The mRNA expression of the wild‐type *TMC1* and Bth *TMC1*. Reproduced with permission.^[^ ^61^ ^]^ Copyright 2016, The Authors. c) Schematic diagram of the in vivo genome editing using the AAVSaCas9–KKH–Myo6‐g2 system. Reproduced with permission.^[^ ^63^ ^]^ Copyright 2021, The American Society of Gene and Cell Therapy.

Gene replacement is a strategy that deliver normal gene into cells to provide the functional protein, which is the most common strategy of gene therapy and have been widely studied in the treatment of recessive hearing loss. In these works, adeno‐associated virus (AAV) is a typical tool as the carrier for DNA delivery. The first work was reported in 2012, in which the *VGLUT3* knockout mice displayed good hearing recovery.^[^ ^59^ ^]^ Recently, researchers used AAV serotype 1 (AAV1) and Anc80L65 to carry human otoferlin (*hOTOF*) coding sequence and form the *h* *OTOF* transgene vector for human hearing loss treatment^[^ ^12b,c^ ^]^ (**Figure** **5**a). They designed a dual‐carrier strategy that used two AAV carriers to carry the *hOTOF* gene, and then delivered them into the inner ear of patients (5.6 × 10^11^ or 1.12 × 10^12^ vg).^[^ ^12c^ ^]^ By this treatment, the hearing of several patients was recovered. These studies first explored the safety and efficiency of gene therapy, opening a new era in clinical treatment of hearing loss.

Gene suppression therapy is realized by regulate the expression of RNA rather than DNA, and it can be divided into two categories: antisense oligonucleotides (ASOs) and RNA interference (RNAi). ASOs can bind with the complementary RNA sequences to upregulate, downregulate, or alter the isoform of proteins. With these features, ASOs can tune the expression of mutant genes, and this method has shown potential in the treatment of usher syndrome (USH). For example, Lentz et al. proved that the applications of ASOs could correct the expression of *USH1C* in inner ear, improving the sensitivity and recovering hearing thresholds.^[^ ^60^ ^]^ By contrast, RNAi is realized by neutralizing mRNA via short interfering RNAs and microRNAs (miRNAs). This technology has been employed to complement with the mutated *TMC1* transcript so as to prevent hearing loss of *TMC1*‐Bth mutant mice (Figure 5b).^[^ ^61^ ^]^

Gene editing is a technology that can disrupt or correct the gene at the specific position by adding, deleting, or replacing the bases of the DNA molecules. Especially, clustered regularly interspaced short palindromic repeat (CRISPR)/CRISPR‐associated proteins (CRISPR–Cas) and base editing have been widely explored in hearing loss treatment.^[^ ^62^ ^]^ Cas9 nucleases can insert or delete the bases to efficiently treat the dominant‐negative alleles and gain functions. Xue et al. using AAV–PHP.eB to deliver *Staphylococcus aureus* Cas9 (SaCas9–KKH)–single‐guide RNA (sgRNA) complexes to improve hearing loss (1.865 × 10^10^ genome copies per cochlea) (Figure 5c).^[^ ^63^ ^]^ In their work, the auditory function of mice was rescued up to 5 months after treatment. Base editors have also been explored in hearing loss treatment. Liu and co‐workers used dual AAVs to package cytosine base editors to repair the recessive *TMC1* mutation of Baringo mice (6.5 × 10^8^ and 8.3 × 10^8^ vg for high dose and 3.1 × 10^7^ and 4.2 × 10^8^ vg for low dose).^[^ ^64^ ^]^ This method reached a reversion rate of 51% of the mutation, and rescued the low‐frequency hearing for 4 weeks after treatment.

Although gene therapy has shown huge potential and exciting results in clinic, it still meet some limitations. The transduction efficiency of the virus vectors in the HCs is not ideal, and that in other types of cells within the inner ear is not clear. The safety of the virus vectors is another issue, including their immune response and off‐target function. In addition, the longevity of therapeutic effects should also be considered because the current methods are hard to last for a long time and thus affect the therapeutic effects.

### 3.4 Exos

Exos are small extracellular vesicles (EVs) released by cells with diameters 30–150 nm.^[^ ^65^ ^]^ They are capable of carrying many biologically active molecules, such as proteins and RNA, and play complex and delicate roles in tissues. On the one hand, Exos can interact directly with damaged cells to promote repair and regeneration by delivering bioactive molecules such as growth factors and cytokines. On the other hand, Exos can also enhance the overall defense of tissues by regulating intercellular signaling. In recent years, Exos also play a nonnegligible role in protecting against hearing loss in the inner ear.^[^ ^66^ ^]^

**Figure 6:**
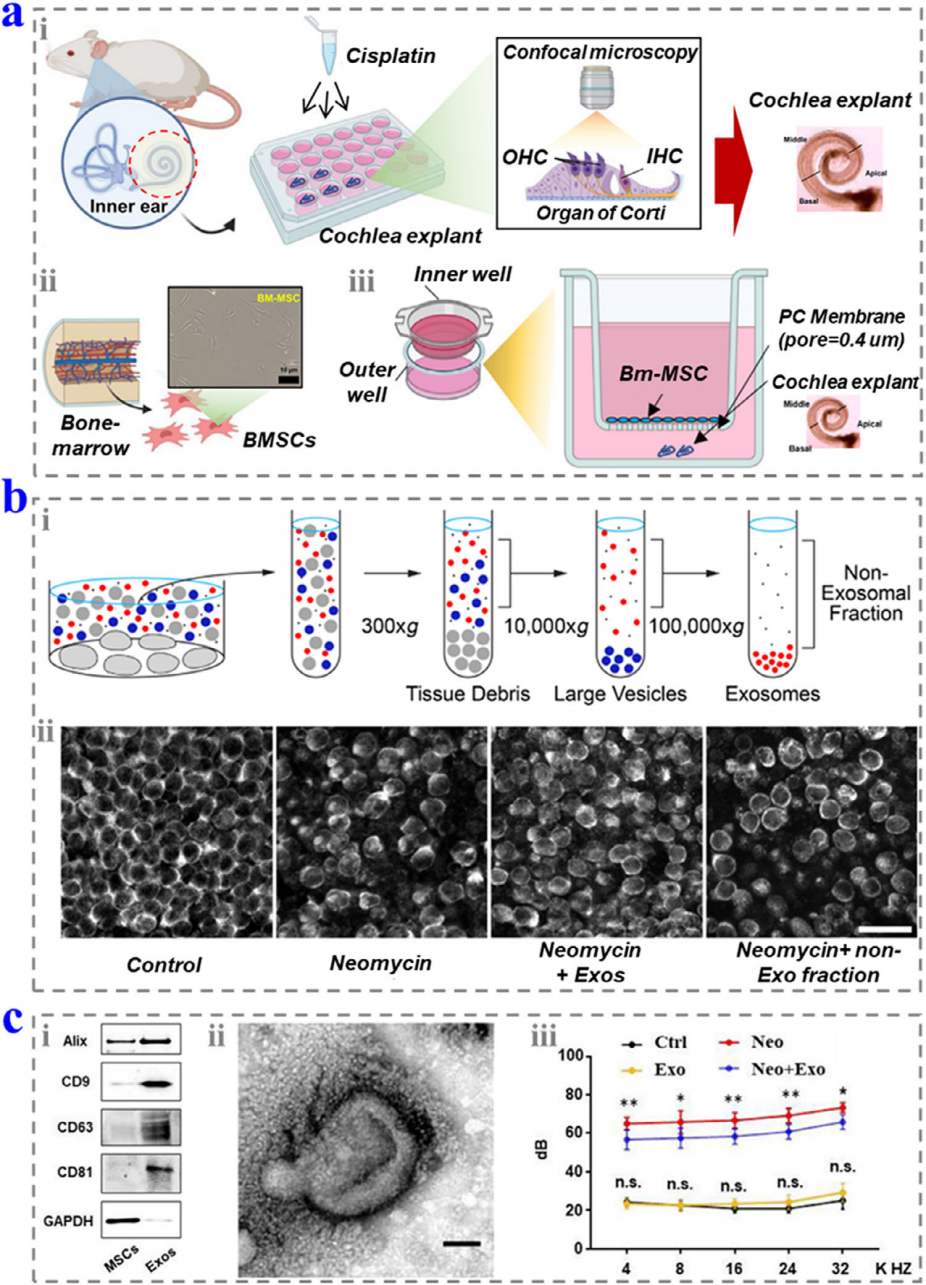
a) Schematic diagrams of i) the toxicity in cochlear explants, ii) isolation of BMSCs, and the coculture study of BMSCs and cochlear explants. Reproduced with permission.^[^ ^68^ ^]^ Copyright 2021, Elsevier. b) i) Schematic diagram of isolating exosomes from the culture medium. ii) The utricles with different treatments for 24 h in neomycin. Reproduced with permission.^[^ ^69a^ ^]^ Copyright 2020, American Society for Clinical Investigation. c) i) The analysis of the biomarkers of Exos. ii) The TEM image of the Exo. iii) ABR test of the mice in different groups. Reproduced with permission.^[^ ^66a^ ^]^ Copyright 2024, The Authors.

Due to the powerful immunomodulatory effects, the EVs derived from human mesenchymal stem cells (MSCs) from the umbilical cord and bone marrow (BMSC–EVs) (1 µL) both significantly protected the cells across species barriers and showed good alleviation effect to the HC loss induced by noise.^[^ ^67^ ^]^ The pretreatment of MSCs could improve their protective efficiency. In addition, Exos can deliver signals and open the route of noncellular autonomous protection of HCs. Park et al. proved that the BMSC‐derived Exos that contained heat shock protein 70 (HSP70) could treat cisplatin‐induced ototoxicity in the coculture model (2.48 × 10^10^ particles mL^−1^ in culture medium) (**Figure** **6**a).^[^ ^68^ ^]^ Breglio et al. found that the HSP70‐loaded Exos could be released by the inner ear tissues that responded to the heat stress. Moreover, the Exos carried with HSP70 could improve the survival rate of HCs exposed to the neomycin by the interaction with the toll‐like receptor 4 (TLR4) (Figure 6b).^[^ ^69^ ^]^ Liu et al. studied the protective role of the MSC‐derived Exo, and found that they play an important role in increasing autophagic activity, which improves the survival of the cells treated by neomycin (Figure 6c).^[^ ^66a^ ^]^ Exos can also associate with AAV to realize gene therapy. Compared with the conventional AAV that was hard to transduce IHCs, the Exo–AAV showed good efficiency in transducing to all HCs (more than 95% IHCs and about 50% OHCs in the best cases).^[^ ^70^ ^]^

Exos have great potential and broad application prospects as a new treatment strategy for sensorineural hearing loss due to their low immunogenicity and good biocompatibility. However, Exos therapy also faces some challenges. First, the isolation and purification techniques need to be further improved to enhance their purity and activity. Second, the therapeutic efficacy and mechanism of Exos need to be verified and clarified through more studies. Finally, for clinical applications, large‐scale production and storage should be addressed.

### 3.5 Other Drugs

Apart from antioxidant drugs, anti‐inflammatory drugs, and gene drugs, there are many other types of drugs, which can play roles such as regulating apoptosis and protecting neurons, are also involved in hearing loss therapy (Table 1).

**Figure 7:**
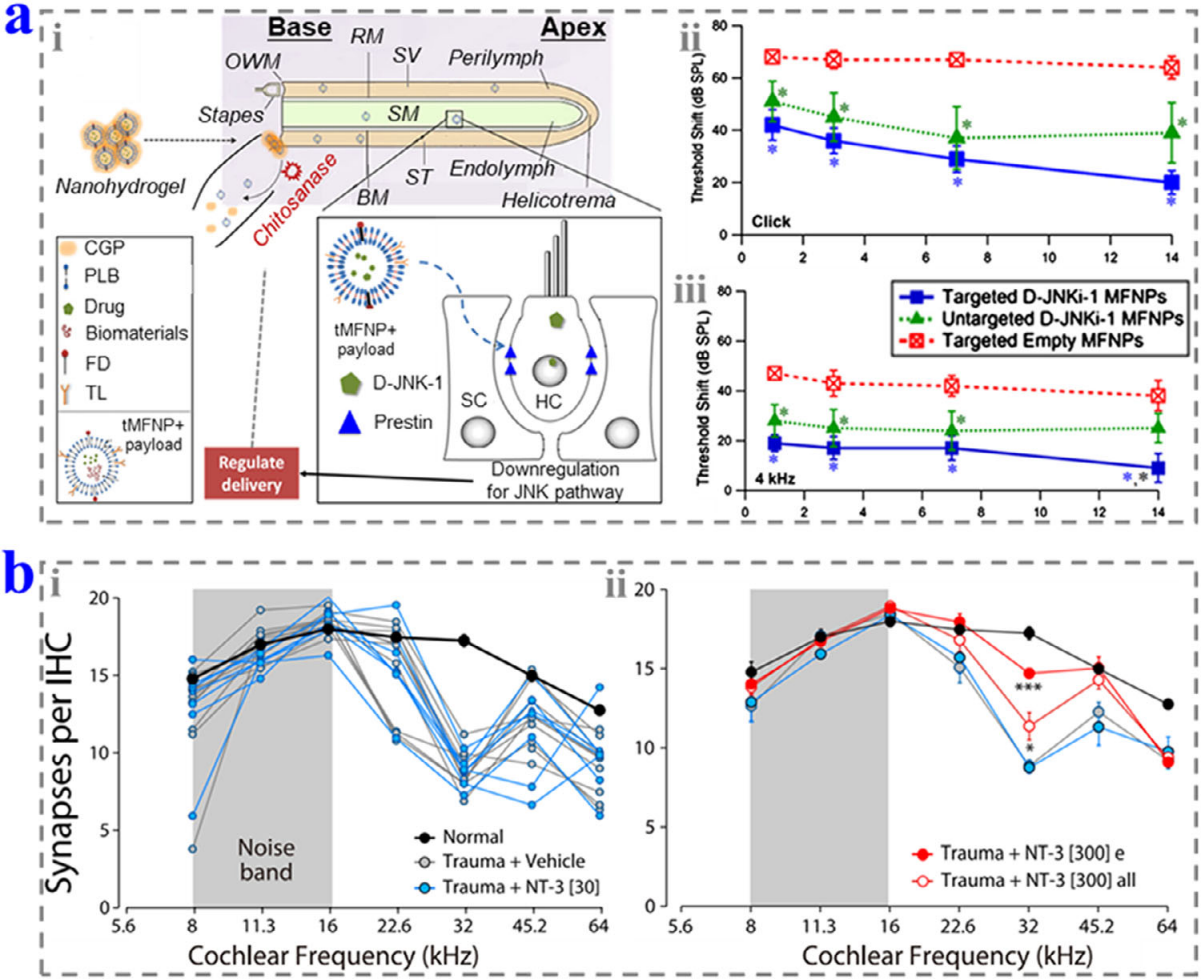
a) i) Schematic diagrams of the targeted nanohydrogel‐based drug delivery system. ii, iii) The hearing threshold shifts of the different groups response to click (ii) and 4 kHz (iii). Reproduced with permission.^[^ ^71b^ ^]^ Copyright 2018, Elsevier. b) i) The average normal synaptic counts of the animals in different groups. ii) The average synaptic counts in each groups. Reproduced with permission.^[^ ^78^ ^]^ Copyright 2016, Springer Nature. OWM: oval window membrane; RM: Reissner's membrane; SV: scala vestibuli; SM: scala media; ST: scala tympani; BM: basilar membrane; PLB: phospholipid bilayer; FD: fluorescent dye; TL: targeting ligand; HC: hair cell; SC: supporting cell.

Caspases play important roles in regulating apoptosis. Typically, caspase‐3 and caspase‐9 can be initiated by cisplatin and induce the apoptosis of HCs and cochlear cell lines, while the intracochlear prefusion of their inhibitors performs good protective function against cell death and hearing loss. The administration of JNK inhibitors, such as CEP‐1347 (KT7515) and D‐JNKi‐1 (AM‐111), also can intervene the activation of caspases and help to protect hearing.^[^ ^71^ ^]^ Li and co‐workers designed targeted nanohydrogel (0.2 µL per ear) to deliver D‐JNKi‐1 to OHCs to protect hearing from NIHL (**Figure** **7**a).^[^ ^71b^ ^]^ p53 is another apoptosis initiator that initiates apoptotic signal under the trigger of stress signal.^[^ ^72^ ^]^ Therefore, delivering p53 inhibitor into inner ear to relieve the stress signal is feasible to improve HC damage.^[^ ^73^ ^]^ For example, the applying of pifithrin‐α (100 µm for organotypic culture), the p53 inhibitor, showed a good protective role on the cochlear organotypic model, which was attributed to the reductive expression of p53, caspase‐1, and caspase‐3.^[^ ^74^ ^]^ The further intratympanic drug administration indicated that the pifithrin‐α could protect hearing without compromising the efficiency of cisplatin. Besides, the study on kenpaullone (310 µm) showed that it could serve as an inhibitor of cyclin‐dependent kinase 2 (CDK2) to protect cells in inner ear from damage and thus, reduced the threshold shift caused by cisplatin and noise.^[^ ^75^ ^]^

The function loss of neurons in the inner ear affects the signal transduction, thus causing hearing loss. Neurotrophic factors can promote the growth, development, and functional integrity of neurons, which further protects SGNs and promotes their regeneration.^[^ ^76^ ^]^ It has been proven that the application of brain‐derived neurotrophic factor (BDNF) and neurotrophin‐3 (NT‐3) could reduce synaptopathy and recover high‐frequency hearing after exposing in noise.^[^ ^77^ ^]^ Furthermore, Suzuki and co‐workers found that NT‐3 (30 or 300 ng) could promote the regeneration of the synapses after noise exposure (Figure 7b).^[^ ^78^ ^]^ Meanwhile, the application of agonist of cannabinoid 2 receptor (CB2R), transtympanic administration of (2‐methyl‐1‐propyl‐1*H*‐indol‐3‐yl)‐1‐naphthalenylmethanone (JWH015), could also reduce the loss of ribbon synapses after cisplatin dose and prevent hearing loss.^[^ ^79^ ^]^ In addition, the exceed glutamate release is another reason for neuronal cell death, which causes the excessive and long‐time activation of the glutamate receptors. Duan and co‐workers presented a gelfoam containing the antagonist of glutamate receptors, caroverine (24 µg for low dose and 192 µg for high dose), reduced the threshold shift caused by noise successfully.^[^ ^80^ ^]^

## 4 Routes for Emerging Inner Ear Drug Delivery Systems

The emerging inner ear drug delivery is mainly realized by the local delivery systems, including tympanic administration or intracochlear administration, due to the high efficiency and low side effects.^[^ ^105^ ^]^ Tympanic administration is often inner ear‐noninvasive, and RWM and OWM are the channels for drug transmission.^[^ ^106^ ^]^ By contrast, intracochlear administrations, including cochlear apex delivery and CI delivery, are invasive to the inner ear and are realized by surgery operations (**Table** **2**).

**Table 2:** Typical administration strategies. Abbreviations. HGC: hexanoyl glycol chitosan; DEX: dexamethasone; NAC: *N*‐acetylcysteine; ATX–LPN: astaxanthin‐loaded polymer–lipid hybrid nanoparticles; GelMA: gelatin methacryloyl; Alg: alginate; ALA: alpha‐lipoic acid; NR: not reported; DNQX: 6,7‐dinitroquinoxaline‐2,3‐dione; AP: artificial perilymph; CI: cochlear implant; DEX: dexamethasone; SGN: spiral ganglion neuron.

Administration strategies	Carriers	Materials	Dosage	Objects	Refs.
Intratympanic injection	Nanoparticles	ATX–LPN, 132.28 ± 1.37 nm, 1 mg mL^−1^	6 µL	Guinea pigs	[122]
	Microparticles	Magnetic GelMA–Alg hydrogel microrobots containing ALA, 71.6 ± 3.9 µm	NR	Mice treated with 25 mg kg^−1^ cisplatin	[128]
	Injectable hydrogel	HGC containing 5 mg mL^−1^ DEX	100 µL	Guinea pigs	[134]
	Injectable hydrogel	P407 hydrogel containing 4% NAC	50 µL	Guinea pigs	[143]
Intracochlear injection	Micropump	Four selectable ports connected to: i) a large fluidic capacitor used for fluid storage (DNQX in AP, 238 µL, 300 µm); ii) an outlet that connects to the drug‐delivery target; iii) the outlet from an integrated drug reservoir; iv) the inlet to the integrated drug reservoir	1 µL, 0.2 µL min^−1^	Guinea pigs	[114b]
	CI	CI loaded with DEX (1% or 10%)	–	Gerbils	[118]
	Nanoparticles	Calcium phosphate nanoparticles loaded with nucleic acids, about 160 nm, 0.9–1.3 × 10^10^ particles mL^−1^	NR	SGN cells	[126]
	Microparticles	6–8 µm, ≈7 × 10^7^ particles mL^−1^	5 µL	Guinea pigs	[131a]

### 4.1 Intratympanic Administration

**Figure 8:**
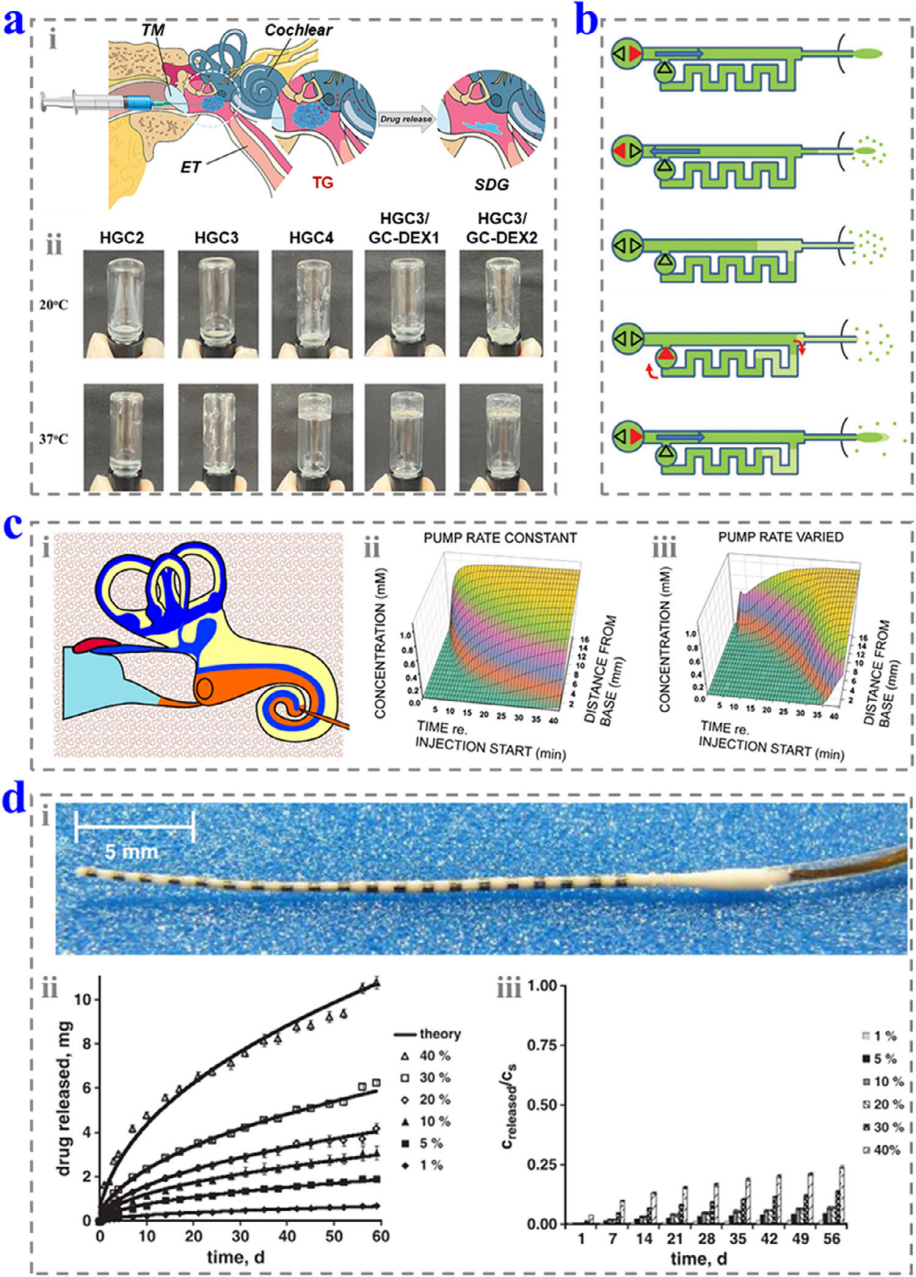
a) i) Schematic diagram of delivering the thermogel formulation via intratympanic administration. ii) The sol–gel transition behavior of the formulation. Reproduced with permission.^[^ ^109^ ^]^ Copyright 2023, The Authors. b) Schematic diagrams of the work mechanisms of the reciprocating microfluidics‐based drug delivery system. Reproduced with permission.^[^ ^115^ ^]^ Copyright 2014, Royal Society of Chemistry. c) i) Schematic diagram of delivering drugs using injection method from cochlear apex. ii, iii) The drug concentration changed along time with a constant injection rate ii) and a variable injection rate (iii). Reproduced with permission.^[^ ^116^ ^]^ Copyright 2016, Elsevier. d) i) Optical image of a CI loaded with 10% DEX. ii, iii) The drug release rate (ii) and the drug saturation of the samples (iii). Reproduced with permission.^[^ ^117^ ^]^ Copyright 2011, Elsevier. TM: tympanic membrane; ET: Eustachian tube; TG: thermogelation; SDG: self‐degradation.

RWM is the most used structure for drug penetration from the middle ear.^[^ ^22^ ^]^ In this system, the drug carriers are delivered into the middle ear, and the released drugs penetrate RWM and reach the inner ear. Since the RWM has semipermeable film properties, it is suitable for delivering cationically charged or lipophilic‐soluble drugs, while it is less effective in the delivery of anionic and water‐soluble drugs. In addition, small molecular‐weight drugs or drug carriers are mainly delivered through diffusion, while large molecular‐weight drugs or carriers require endocytosis and other ways.^[^ ^107^ ^]^ Based on these features, the drugs used for trans‐RWM administration are carefully selected. In addition, to avoid drug loss in the middle ear due to the clearance effect of the Eustachian tube, the drug carriers are often fixed on RWM to acquire higher efficiency. Some delivery systems are designed as microneedles to acquire better tissue adhesion and penetration.^[^ ^108^ ^]^ However, the average thickness and area of RWM in humans are about 70 µm and 2.3 mm^2^, while those are 10–14 µm in rodents and 1.2 mm^2^ in guinea pigs. Therefore, studies on animals may not be suitable for clinical settings. Le et al. designed a self‐degradable prodrug blend thermogel to treat sensorineural hearing loss by intratympanic injection (**Figure** **8**a‐i).^[^ ^109^ ^]^ In the work, DEX was conjugated glycol chitosan to form GC─DEX, and combined with hexanoyl glycol chitosan (HGC) to obtain an injectable formulation. The formulation was solution at a relatively low temperature, while it transformed into hydrogel at the body temperature (Figure 8a‐ii). This feature allows it to be injected into the middle ear easily and has a long‐lasting stability within the tympanic cavity.

Although the stapes footplate covers OWM, it can also be used for inner ear drug delivery due to its semipermeable film properties. It was proven that OWM could deliver 35% of drugs against 57% or 65% of drugs delivered by RWM.^[^ ^110^ ^]^ The fixing of drug carriers may last the drug release for years.^[^ ^111^ ^]^ Nevertheless, it is more suitable for delivering drugs for vestibular diseases rather than sensorineural hearing loss.

### 4.2 Intracochlear Administration

Intracochlear administration is a precise strategy for inner ear drug delivery that avoids systemic targeting and premature systemic clearance.^[^ ^112^ ^]^ It is often realized by catheters directly to the cochlea or the cochleostomy. Besides, sustained drug delivery can be realized by a miniaturized wearable device that contains the drug solution to avoid frequent invasion. In contrast to intratympanic administration, intracochlear administration bypasses the middle ear, allowing the drug to reach the intended site directly and achieve better drug bioavailability and controllable dose.^[^ ^113^ ^]^ In addition, the drugs delivered from the RWM are hard to reach the cochlear apex due to the slow drug diffusion and complex structure, while the drug delivery route from the cochlear apex injects the drugs into the inner ear with precise control. Among all the intracochlear drug delivery strategies, microfluidics‐based drug delivery system has shown many strengths such as the controllable and precise drug dose, wearable and full implantation capability, and thus, showing great potential in practical applications.^[^ ^114^ ^]^ Kim et al. presented a reciprocating microfluidics‐based drug delivery system, which could deliver high‐concentration drug solution into the cochlea and withdraw the low‐concentration solution at sub‐microliter level to realize drug delivery without volume change of the fluid in the inner ear (Figure 8b).^[^ ^115^ ^]^ Lichtenhan et al. studied the drug distribution behavior in the cochlear spiral of cochlear apex injection.^[^ ^116^ ^]^ They found that this method could regulate the drug solution delivery, maintain the drug concentrations, and affect the hearing from the low to high frequency sequentially (Figure 8c). However, this process is highly invasive and needs surgical access to the cochlea, which may cause damage and postoperative complications, including inflammation and protein fouling.

The application of a CI can help to improve the hearing while causing local trauma and inflammation. To relieve these side effects, the implants can be modified with drugs.^[^ ^118^ ^]^ For example, Siepmann and co‐workers doped DEX into the CI.^[^ ^117^ ^]^ As shown in Figure 8d‐i, due to the drug loading, the polymer shows a white color rather than transparent. The drugs could be released from the CI, and it can last for more than 8 weeks (Figure 8d‐ii,iii). Apart from mixing the drugs into the polymer, the drugs can also be loaded on the CI by using femtosecond laser technology. StÖver et al. used the silicone sheet to evaluate the laser parameters first, and then transferred the data to the CI to form the prototypes.^[^ ^119^ ^]^ The prototypes exhibited a satisfactory result in fluid‐based drug delivery.

## 5 Emerging Carriers as the Inner Ear Drug Delivery Systems

### 5.1 NPs

Benefiting from the rapid development of nanotechnology in the drug delivery field, the application of NP‐based inner ear drug delivery systems has gained increasing attention.^[^ ^120^ ^]^ NPs can encapsulate indissolvable drug drugs such as DEX to increase their solubility and thus improve bioavailability while protecting the drug from degradation in the body environment and prolonging its half‐life.^[^ ^121^ ^]^ Besides, the modification of the NPs can improve their tissue penetration and bioavailability, as well as specifically recognize and bind to receptors on the surface of inner ear cells to achieve targeted drug delivery. Therefore, NPs are popular in inner ear drug delivery.

**Figure 9:**
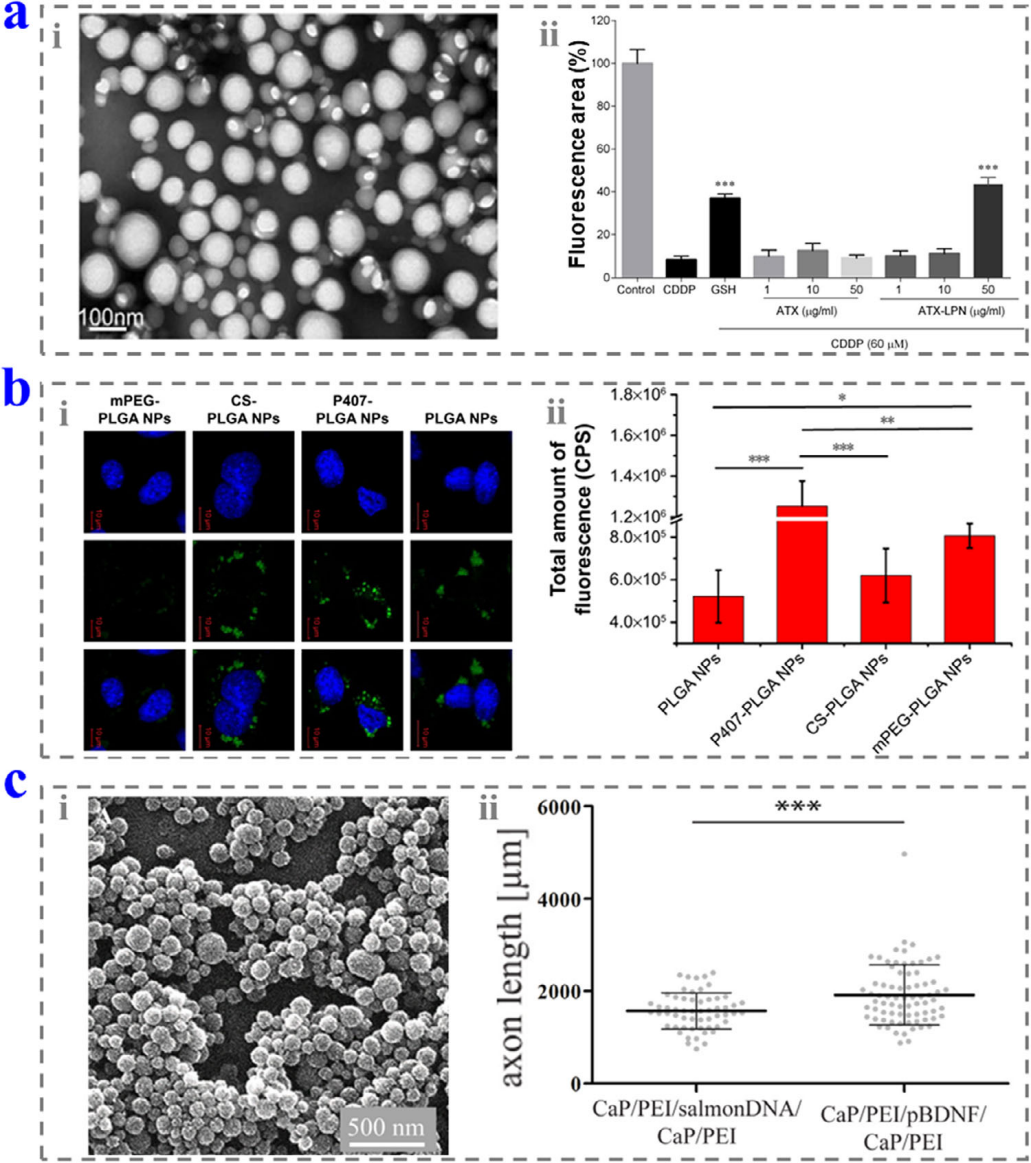
a) i) The SEM image of the ATX–LPN. ii) The protective effect of ATX–LPN. Reproduced with permission.^[^ ^122^ ^]^ Copyright 2020, Springer Nature. b) i) The cell uptake results of the NPs with different modifications. ii) The fluorescence intensity in the cochleae. Reproduced with permission.^[^ ^124^ ^]^ Copyright 2016, The Authors. c) i) SEM image of the CaP NPs. ii) The axon length of the SGNs in different groups. Reproduced with permission.^[^ ^126^ ^]^ Copyright 2021, Elsevier.

Astaxanthin (ATX) is a candidate for oxidative stress treatment because of its unique antioxidant capacity, but it suffers poor solubility. Wu and co‐workers designed a lipid–polymer hybrid nanoparticle (LPN) for ATX loading (**Figure** **9**a).^[^ ^122^ ^]^ The formed ATX–LPN showed good biocompatibility and could significantly affect the generation of cisplatin‐induced ROS. In the RWM administration, ATX–LPN (6 µg) could be delivered into the inner ear and maintain a proper concentration for 24 h. Meanwhile, it could reverse the decrease in the cisplatin‐induced mitochondrial membrane potential and thus rescue the cells from the early stages of apoptosis. Furthermore, it relieved the OHC loss in the animal model by downregulating the expression of caspase 3/9 and cytochrome‐c and upregulating the expression of the antiapoptotic protein Bcl‐2. In the following work, they found that the ROS‐responsive NPs made by ATX and poly(propylene sulfide) could also treat cisplatin‐induced ototoxicity with a satisfactory result.^[^ ^123^ ^]^

The effects of the different modifications on the poly(lactic/glycolic acid) NPs (PLGA NPs) for inner ear drug delivery was explored by Chen and co‐workers.^[^ ^124^ ^]^ They first evaluated the PLGA NPs modified with different molecules (2.5 mg) and found that the modification of Poloxamer 407 (P407) had the best cell uptake efficiency and enriched in the OHCs with a higher level (Figure 9b). Furthermore, they studied the effects of the size, surface charge, and cell‐penetrating peptide modification.^[^ ^125^ ^]^ The results indicated that the NPs with larger sizes had a larger entry amount in the cochleae. The chitosan (CS)‐modified NPs had positive surface charges and the fastest rate to the cochleae, while the NPs modified with P407 showed the largest distribution after 24 h. Besides, the NPs modified with low molecular weight protamine (LMWP) showed excellent properties in cell uptake. Based on these features, they combined the P407–PLGA NPs and LMWP to promote the entry to the Corti and stria vascularis without pathological changes to the cochlear tissues and RWM.

Apart from delivering the NPs to the inner ear by intratympanic injection, the NPs can be integrated with CIs to relieve the inflammation caused by the implanted foreign bodies. Epple and co‐workers coated the CI electrodes with calcium phosphate (CaP) NPs containing DNA by electrophoretic deposition and layer‐by‐layer coating.^[^ ^126^ ^]^ It was found that the coating did not block the signals, while the NPs could be used as the transfection agents to deliver plasmid DNA by the cell uptaking. When the BDNF‐encoding plasmid DNA was loaded, the NPs could promote the secretion of growth factors and axonal growth, thus helping to protect hearing (Figure 9c).

### 5.2 Microparticles

Microparticles have a much larger volume than NPs, and therefore, there are some significant differences between them when they are used for drug delivery.^[^ ^127^ ^]^ Typically, NP‐based drug delivery relies on the NPs crossing the physiological barriers to enter the inner ear. By contrast, microparticles have difficulty crossing these barriers under normal conditions, resulting in therapy by drug release outside the barriers. The larger size of microparticles makes them easy to be operated and observed. Besides, NPs are often combined with solvents or hydrogels, while microparticles can be delivered directly. These features allow microparticles to be suitable candidates for inner ear drug delivery.

**Figure 10:**
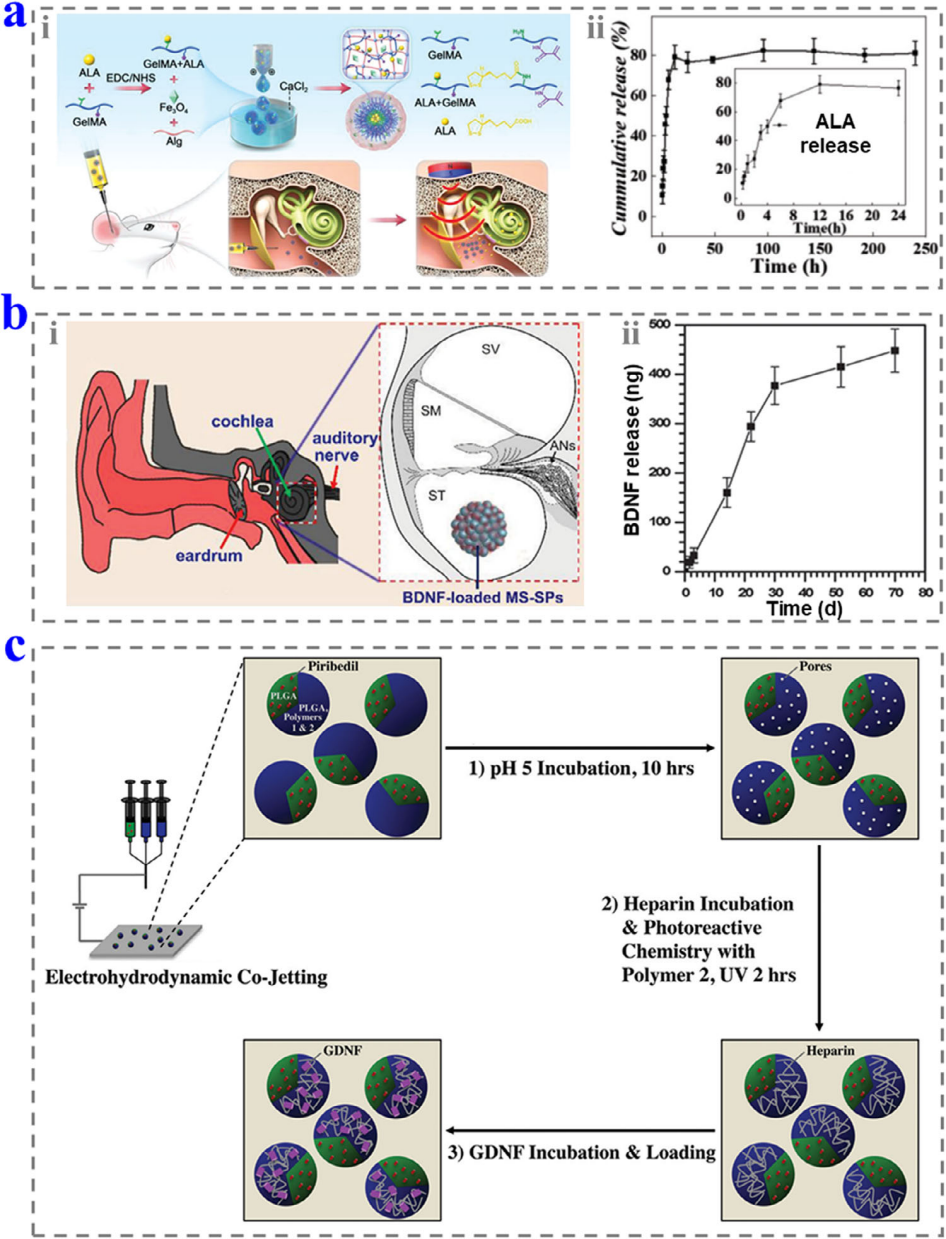
a) i) Schematic diagram of fabricating the hydrogel microparticles and middle ear injection. ii) The drug release capability of the hydrogel microparticles. Reproduced with permission.^[^ ^128^ ^]^ Copyright 2023, Wiley‐VCH. b) i) Schematic diagram of the MSSPs in the inner ear. ii) The drug release capability of the MSSPs. Reproduced with permission.^[^ ^130^ ^]^ Copyright 2014, Wiley‐VCH. c) Schematic diagram of fabricating dual‐compartmental microparticles. Reproduced with permission.^[^ ^131a^ ^]^ Copyright 2015, Wiley‐VCH.

Chen et al. designed a hydrogel microparticle for hearing loss prevention by intratympanic admission.^[^ ^128^ ^]^ They modified the ALA onto the molecule of GelMA first and then mixed it with alginate (Alg) and Fe_3_O_4_ to form a homogeneous solution. By further cutting via microfluidic electrospray and cross‐linking, the drug‐loaded hydrogel microparticles were prepared. The microparticles (containing ALA, 100 mg kg^−1^) could reach the RWM under the guidance of a magnetic field after intratympanic injection, and ALA could be released from the microparticles for a long time to prevent hearing loss (**Figure** **10**a).

In some cases, microparticles can be delivered into the inner ear by surgery operation directly.^[^ ^129^ ^]^ Wang et al. developed a type of mesoporous silica (MS) NP‐based superparticles (MSSPs) by self‐assembly and delivered them (7 particles per ear) into the inner ear for the treatment of hearing loss.^[^ ^130^ ^]^ The MS NPs can effectively load drugs such as BDNF to protect them from the external environment and achieve their release in the inner ear. The results showed that the MSSPs improved the survival of primary auditory neurons, indicating their good protective effect on hearing (Figure 10b).

When the microparticle has a small size (about several micrometers), they can be delivered into inner ear with a large amount, which similar to NPs. Lahann and co‐workers designed dual‐compartmental microparticles as drug carriers for hearing loss treatment.^[^ ^131^ ^]^ In their work, the drug solution channels were cut into droplets and dried fast to form microparticles via microfluidic electrospray technology. The prepared microparticles had good biocompatibility and biodegradation, while the size of 6–8 µm could delay the uptake by macrophages. By further drug‐loading treatment, the microparticles (about 3.5 × 10^5^ particles per ear) could be delivered into inner ear to release the anti‐excitotoxic drugs over a short period to relieve the damage caused by the CI implantation and then release BDNF over a longer period to protect HCs (Figure 10c).

### 5.3 Injectable Hydrogels

Injectable hydrogels show significant advantages as a local drug delivery system in the inner ear.^[^ ^132^ ^]^ Injectable hydrogels have excellent biocompatibility and degradability, and can exist in the middle or inner ear without causing serious immune responses or tissue damage. They have certain viscoelasticity and tissue adhesion properties, which allow them to adhere to the middle ear cavity to avoid the rapid clearance of the Eustachian tube. Besides, hydrogels release drugs continuously and controllably, which ensures the effective drug concentration at the target site for a long time, thus improving the therapeutic effect. Since there are many raw materials can be employed to form hydrogels, the components can be chosen to match the physicochemical properties of drugs and the microenvironment of the middle or inner ear. The typically used hydrogels include natural polymers such as CS, hyaluronic acid (HA), and SF, as well as synthetic polymers such as polyethylene glycol (PEG) and Poloxamer.

**Figure 11:**
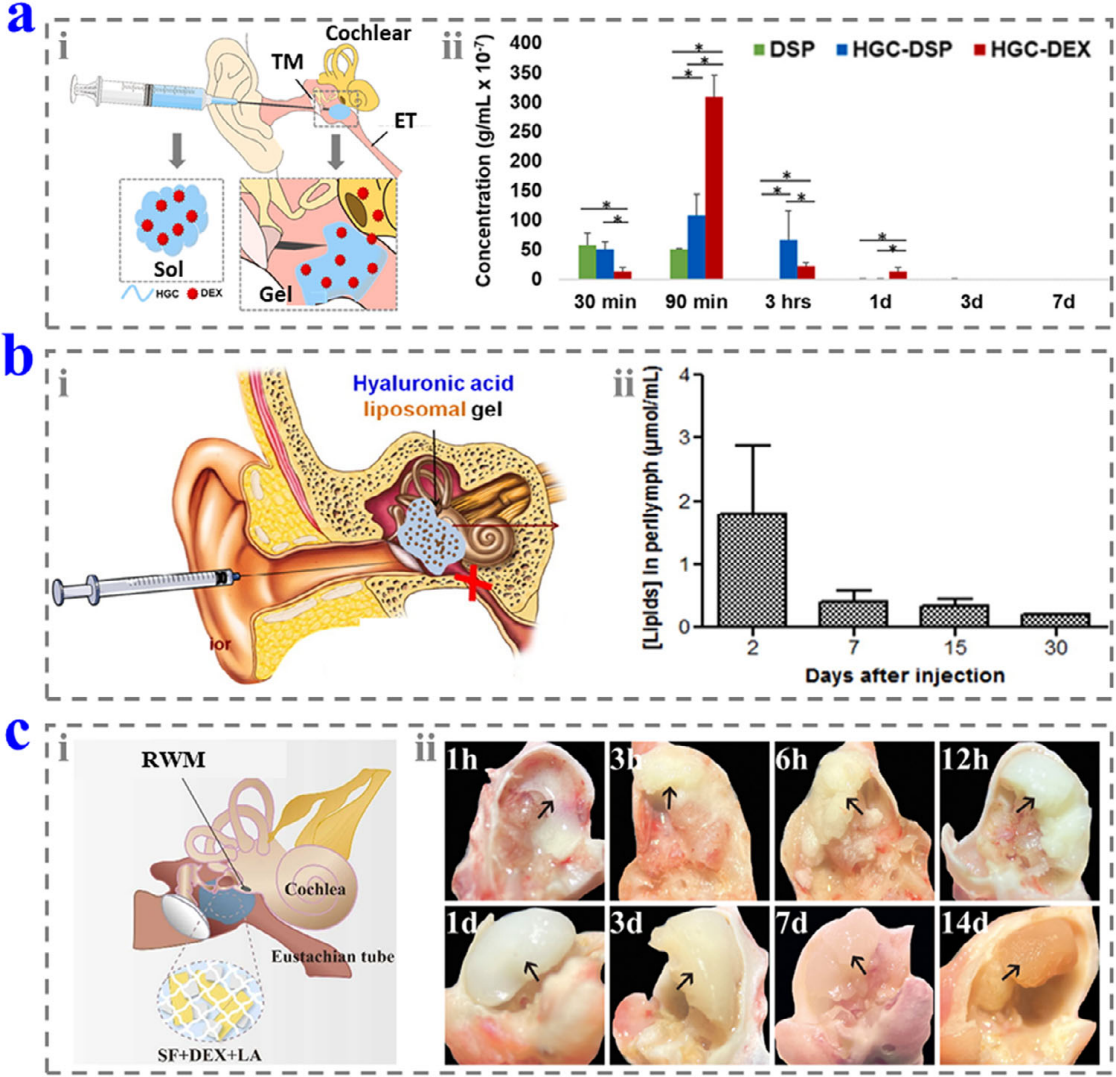
a) i) Schematic diagram of the usage of the HGC–DEX hydrogel. ii) DEX concentration in the intracochlear perilymph of different groups. Reproduced with permission.^[^ ^134^ ^]^ Copyright 2022, Elsevier. b) i) Schematic diagram of the usage of the HA hydrogel containing DEXsp. ii) The drug concentration in the intracochlear perilymph after injection. Reproduced with permission.^[^ ^137^ ^]^ Copyright 2016, Elsevier. c) i) Schematic diagram of using SF + DEX + LA for hearing loss treatment. ii) The photographs of the SF + DEX + LA in the middle ear at different times. Reproduced with permission.^[^ ^139^ ^]^ Copyright 2024, Elsevier. ET: Eustachian tube; RWM: round window membrane.

CS is a natural polysaccharide‐derived cationic polymer and has been widely used in the biomedical field due to its good biocompatibility.^[^ ^133^ ^]^ Moreover, it can be modified to acquire more functions. For example, HGC has amphiphilic and thermal‐responsive properties, and thus, it is possible to increase the solubilization of the hydrophobic DEX (0.5 mg) and perform sol–gel phase change in the middle ear (**Figure** **11**a).^[^ ^134^ ^]^ With these features, the HGC solution containing DEX (HGC–DEX) can be injected into the middle ear to form a hydrogel, and DEX can be released sustainably to the inner ear. Apart from the thermal‐responsive sol–gel phase change, CS solution can also form a hydrogel by the cross‐linking of genipin.^[^ ^135^ ^]^

HA is a natural polysaccharide that widely exists in the body, and therefore, HA‐based hydrogels are biocompatible, biodegradable, and suitable for in vivo applications.^[^ ^136^ ^]^ In Bochot and co‐workers’ work, the HA hydrogel was employed to load liposomes containing the dexamethasone sodium phosphate (DEXsp) (0.5 or 1.5 mg) and then injected into the middle ear (Figure 11b).^[^ ^137^ ^]^ The injected hydrogel not only had no negative effect on the hearing thresholds but also released the liposomes to the inner ear. Moreover, the liposomes could be detected in the perilymph after 30 days, indicating that the hydrogel had a long time to perform therapeutic effects.

SF is another natural protein suitable for tissue engineering due to its excellent biocompatibility, low immunogenicity, and good biodegradation.^[^ ^138^ ^]^ Diao and co‐workers developed a SF hydrogel containing DEX and ALA (SF + DEX + LA), and injected it into the middle ear of guinea pigs (0.15 mL per ear, DEX and LA were 10 and 100 mg mL^−1^, respectively.)^[^ ^139^ ^]^ The issues showed good morphology and no obvious inflammatory response after hydrogel injection, and the hydrogel could remain in the inner ear for more than 14 days (Figure 11c). Thus, the hydrogel is possible for practical application in hearing loss treatment.

**Figure 12:**
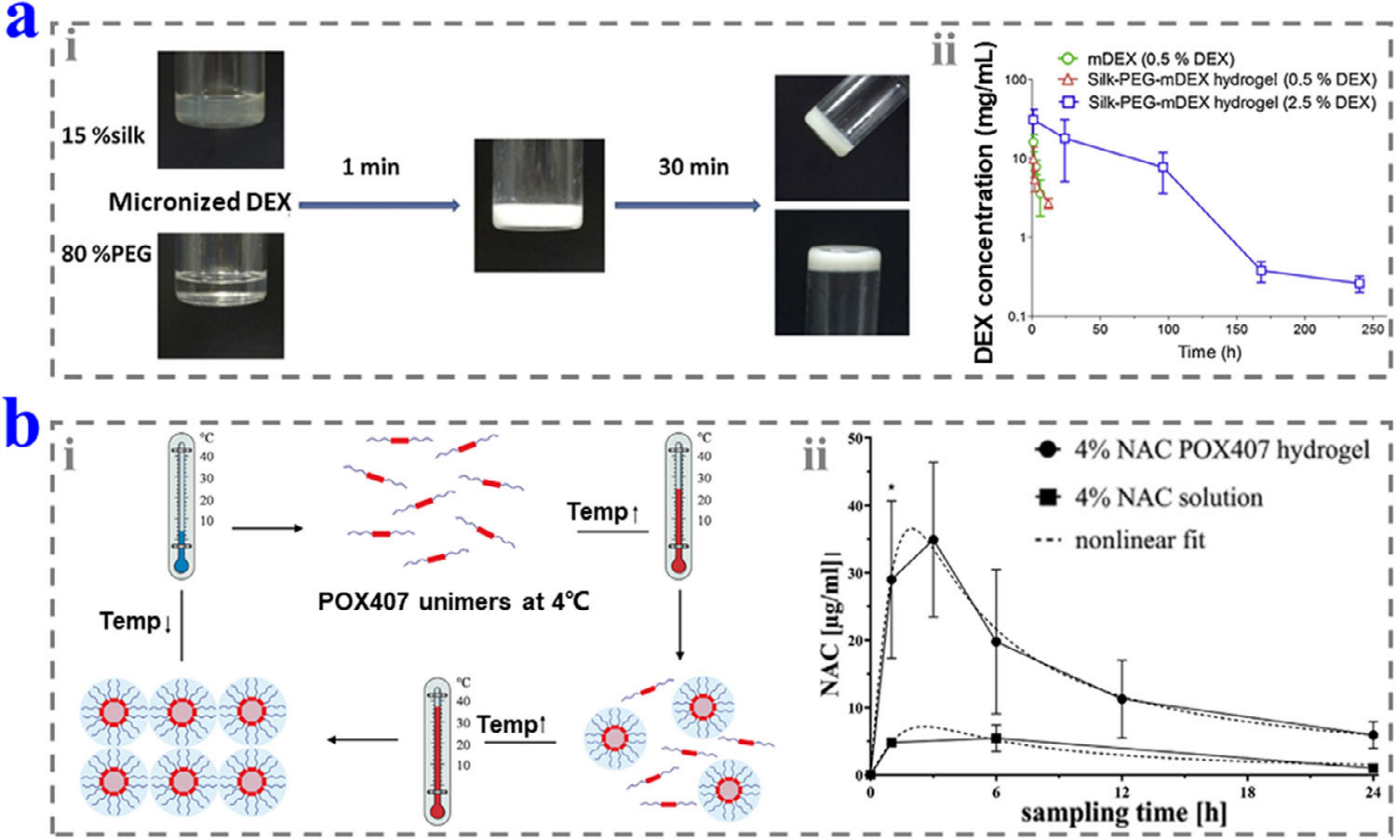
a) i) The formation of the SF–PEG hydrogel. ii) mDEX pharmacokinetics in the inner ear of the SF–PEG–mDEX hydrogel and mDEX solution. Reproduced with permission.^[^ ^141^ ^]^ Copyright 2016, Elsevier. b) i) The thermal‐sensitive properties of P407. ii) The NAC pharmacokinetics in the inner ear of NAC‐containing P407 hydrogel and NAC solution. Reproduced with permission.^[^ ^143^ ^]^ Copyright 2020, Elsevier.

PEG has been widely used in the biomedical field, especially in drug delivery and tissue engineering due to its good biocompatibility, stability, and tissue penetration. Although PEG cannot form hydrogel without modification, it can mix with SF to form an injectable hydrogel (SF–PEG).^[^ ^140^ ^]^ In Wang and co‐workers’ work, the micronized DEX (mDEX) (10 µL, 0.5% or 2.5% w/v) was encapsulated in the SF–PEG hydrogel and then delivered into the inner ear of guinea pigs (**Figure** **12**a).^[^ ^141^ ^]^ When the content of mDEX was lower than 5% w/v, it was dispersed in the solution evenly and had no significant influence on the gelation of the hydrogel. It was shown that the hydrogel containing 2.5% w/v could be injected onto the RWM and deliver DEX into the inner ear to maintain a concentration of 100 ng mL^−1^ for at least 10 days while the free mDEX could reach this concentration for only less than 12 h. Moreover, the hydrogel degraded completely in 21 days without side effects.

Poloxamers are block copolymers of polyoxyethylene and polyoxyethylene and possess good biocompatibility and unique physicochemical properties. For example, OTO‐104 hydrogel was employed to deliver mDEX into the inner ear of the guinea pig and sheep models and maintain the drug concentration for at least 1 and 3 months, respectively. P407 solution is flowing at a low temperature, while it forms a hydrogel when the temperature rises to 37 °C.^[^ ^142^ ^]^ This unique feature allows it to store drugs and release them to the inner ear. Gausterer et al. employed the thermoreversible P407 hydrogel to load NAC (50 µL, 4% NAC/20% POX407 hydrogel) to relieve the hearing loss caused by cisplatin.^[^ ^143^ ^]^ As shown in Figure 12b, the P407 hydrogel could maintain the concentration of the drug in the inner ear better compared with the free NAC solution.

## 6 The Commercialization and Industrialization of Current Therapeutic Platforms

In clinic, some drugs have been employed for relieving hearing loss treatment. Glucocorticoids (typically: dexamethasone solution and methylprednisolone tablets) are mainly used drugs for the sudden sensorineural hearing loss due to their excellent role in anti‐inflammation and immunosuppressive. Small molecular antioxidants (typically: vitamin C, vitamin E, and coenzyme Q10), can relieve the oxidative stress of inner ear cells and protect auditory cells, and thus promote hearing recovery. Vasodilator substances, such as alprostadil injection, ginkgo biloba extract injection, and ginkgo biloba leaf extract tablets, have also been utilized for improving the blood circulation of cochlear to promote hearing recovery. Neurotrophic drugs, including methylcobalamin dispersible tablets, citicoline sodium capsules, methylcobalamin injection, can promote the growth and repair of nerve cells, improve the function of auditory nerve, and enhance their ability to resist damage by providing essential nutrients. Recently, Pedmark, a formulation of STS, has been approved by US Food and Drug Administration for the treatment of children's hearing loss caused by cisplatin during chemotherapy for nonmetastatic solid tumors. It is the first drug for preventing hearing loss caused by ototoxic drugs, while it is not suitable for most patients. In addition, as drug‐free treatment, hyperbaric oxygen therapy has also been applied in clinic for sudden hearing loss. The oxygen content in blood can be increased under high pressure, thereby improving microcirculatory hypoxia in patients. Although there are many strategies that have shown potential in hearing loss treatment, seldom of them have shown specificity.

## 7 Ongoing Clinical Applications

There are many clinical trials that are performing, and some of them are promising in further clinical applications.^[^ ^144^ ^]^ Atorvastatin has shown good effectiveness on preventing hearing loss of the cisplatin‐treated patients who suffered head and neck cancer (NCT03225157).^[^ ^145^ ^]^ Furthermore, a Phase III trial (NCT04915183) is performing to study the protection extent of it in the patients. The studies on reprograming supporting cells has also been explored. FX‐322 (CHIR99021 and valproic acid) showed good efficiency in promoting HC generation and the conversion of supporting cells to HCs.^[^ ^146^ ^]^ The trial (NCT03616223) showed that the it had good safety and tolerability, and some patients displayed a significant hearing improvement.^[^ ^147^ ^]^ In the aspect of improving glutathione‐like antioxidant activity, the ebselen formulation, SPI‐1005, has been presented to evaluate its role against NIHL (NCT01444846)^[^ ^148^ ^]^ and tobramycin (NCT02819856)‐ or cisplatin (NCT01451853)‐induced ototoxicity. It was found that the 400 mg twice daily of SPI‐1005 was a safe dose and effective in prevent NIHL.^[^ ^148^ ^]^ ORC‐13661 is a drug derived from PROTO‐1 and can block the mechanoelectrical transducer channel of HCs to reduce the entry of ototoxic drugs to realize hearing protection.^[^ ^149^ ^]^ The clinical trial (NCT05730283) of ORC‐13661 is performing for evaluating its protective role in amikacin‐induced hearing loss of patients suffered nontuberculous mycobacterial infections. SENS‐401 is a new molecule that can inhibit the hyperactivation of the 5‐hydroxytryptamine3 receptor and thus, preventing the neuronal cells from excitotoxic death. It showed good protective function in rats with cisplatin administration, and the Phase I study (NCT03071003) indicated it has good safety and tolerance. Furthermore, the Phase IIa study (NCT05628233) was initiated to study its efficacy on hearing protection of cisplatin‐induced ototoxicity in patients with cancer. Some clinical trials focus on the study of γ‐secretase inhibitor, including PIPE‐505 (NCT04462198) and LY3056480 (ISRCTN59733689), to inhibit Notch signaling. However, although LY3056480 showed good safety and tolerability, it did not meet the primary endpoint.^[^ ^150^ ^]^

## 8 Challenges and Perspective

Traditional hearing recovery strategies mainly are CIs and systemic drug administration. Compared with emerging inner ear drug delivery platforms, there are some similarities and differences. The similarities between traditional hearing recovery strategies and the current inner ear drug delivery platforms are mainly used as drug and administration strategies for novel bioactive agents. Most drugs used in emerging drug delivery platforms, such as glucocorticoids and vitamins, have already been used in clinics. In some cases, the delivery of novel bioactive agents, including NPs, RNA, and exosomes, has been realized using traditional systemic administration. Some of the emerging inner ear drug delivery platforms have developed new administration strategies, including cochlear administration and intratympanic injection, to improve drug delivery efficiency or employ novel drug carriers to deliver drugs sustainably. Cochlear administration is often performed along with CI implantation surgery; it can deliver drugs into the inner ear precisely but carries a high risk of invasion. Therefore, intratympanic injection is the main novel drug delivery route that compromises drug delivery efficiency and invasion. Novel drug carriers, such as injectable hydrogels, NPs, and nanoparticles, can store drugs within them and deliver them into the inner ear for a relatively long time, which avoids the frequent dosing that traditional administration requires.

Owing to its inaccessible location, complex structure, and low blood flow, the inner ear presents obvious difficulties in drug delivery. Although many drug delivery systems can deliver drugs precisely to the inner ear, they face several problems. First, most of these procedures are invasive. Cochlear drug delivery is a highly invasive procedure that disrupts the structure of the inner ear and, if not performed properly, can lead to severe hearing loss, tinnitus, vertigo, and other complications. In addition, intracochlear drug delivery must be precise because if the drug concentration is too high or the drug itself is ototoxic, it can cause irreversible damage to the inner ear tissue. Although intratympanic delivery is a noninvasive method to the inner ear, it usually involves puncturing the tympanic membrane, resulting in tympanic membrane defects, which can also cause ear discomfort, impaired hearing, and even middle ear infection and inflammation. Therefore, intracochlear drug delivery should be performed simultaneously with cochlear implant surgery to avoid additional damage to the cochlear structure. In addition, the development of injectable hydrogels with better shear‐thinning properties is expected to enable tympanic drug delivery through smaller needles, thereby reducing eardrum damage.

Second, the stability of the drug release process is an important challenge. Most drug delivery systems reported thus far suffer from the problem of initial drug burst release. An excessively high drug concentration may cause toxic side effects, whereas an excessively low drug concentration may fail to achieve these therapeutic effects. Therefore, novel drug delivery systems are anticipated to be developed with some gating effects to avoid burst release while ensuring that the drug remains at a high concentration in the later phase.

Improving the stability and efficacy of drugs is another challenge for drug delivery systems. Although many small‐molecule antioxidant drugs or proteins have been validated to have good therapeutic effects, their in vivo application can include challenges, such as denaturation, and thus loss of efficacy. This can be addressed in two ways. On the one hand, drug delivery systems should be finely designed to avoid the action of oxidative substances or enzymes, etc., on the drugs; on the other hand, novel drugs with good stability and can ignore the unfavorable factors in the microenvironment, such as nanozymes, can be developed to achieve good therapeutic effects.

Furthermore, there are significant differences between the physiological structures of humans and animals, and the results obtained from animal experiments may not be consistent with those of humans. For example, as mentioned above, the thickness of the RWM in humans is much greater than that in rodents. Therefore, drugs with good therapeutic effects in animal experiments may not be able to penetrate the RWM in humans. In addition, the ossicle bone in humans is much thicker than that in guinea pigs, so it is very difficult for drugs to penetrate through the ossicle bone and reach other tissues in the human body, which is possible in the guinea pig's body. Therefore, when developing drug delivery systems, it is necessary to consider physiological differences between animals and humans.

Finally, with the continuous development of science and technology, artificial intelligence (AI) technology has made great progress and has shown significance in healthcare. The full integration of AI with drug delivery systems is expected to help design novel drug delivery systems, develop new delivery routes, and select the best strategy from a number of delivery methods, thereby achieving precise and personalized treatment.

## 9 Conclusion

Therapy for inner ear hearing loss is of great importance, and appropriate drug delivery systems suitable for the inner ear structure and microenvironment are important in this field. In this review, we introduce the structure of the inner ear, drug types, drug delivery routes, and carriers; summarize the challenges faced; and propose possible solutions. We hope that this review will arouse the interest of more researchers and promote further development of inner ear drug delivery systems.

## Conflict of Interest

The authors declare no conflict of interest.

## Author Contributions

X.M., Z.X., and H.R. contributed equally to this work. K.C., H.Z., and H.W. conceived the idea. X.M., Z.X., H.R., and H.Z. wrote the paper. H.Z. and H.W. revised the paper.
